# A quantitative description of Ndc80 complex linkage to human kinetochores

**DOI:** 10.1038/ncomms9161

**Published:** 2015-09-08

**Authors:** Aussie Suzuki, Benjamin L. Badger, Edward D. Salmon

**Affiliations:** 1 Department of Biology, University of North Carolina at Chapel Hill, Chapel hill, North Carolina 27599, USA

## Abstract

The Ndc80 complex, which mediates end-on attachment of spindle microtubules, is linked to centromeric chromatin in human cells by two inner kinetochore proteins, CENP-T and CENP-C. Here to quantify their relative contributions to Ndc80 recruitment, we combine measurements of kinetochore protein copy number with selective protein depletion assays. This approach reveals about 244 Ndc80 complexes per human kinetochore (∼14 per kinetochore microtubule), 215 CENP-C, 72 CENP-T and only 151 Ndc80s as part of the KMN protein network (1:1:1 Knl1, Mis12 and Ndc80 complexes). Each CENP-T molecule recruits ∼2 Ndc80 complexes; one as part of a KMN network. In contrast, ∼40% of CENP-C recruits only a KMN network. Replacing the CENP-C domain that binds KMN with the CENP-T domain that recruits both an Ndc80 complex and KMN network yielded functional kinetochores. These results provide a quantitative picture of the linkages between centromeric chromatin and the microtubule-binding Ndc80 complex at the human kinetochore.

## Abstract (web summary)

The Ndc80 complex acts as an interface between kinetochores and spindle microtubules, and is recruited to the kinetochore by several different proteins. Suzuki *et al*. use protein counting techniques and RNA interference to quantify the interaction stoichiometries within this intricate cellular structure.

## Graphical abstract

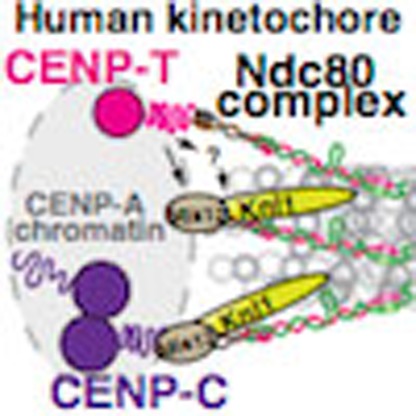

The Ndc80 protein complex (Ndc80c) has a number of critical functions within the outer kinetochore needed for accurate chromosome segregation. These functions include: robust end-on attachment to the plus ends of spindle microtubules (MTs) to form kinetochore MTs (kMTs) that mechanically link kinetochores to spindle poles; force generation during plus-end depolymerization and polymerization; phosphorylation-dependent correction of errors in kMT attachment; and control of the spindle assembly checkpoint^1^,^2^,^3^,^4^. Ndc80c is a heterotetramer of Ndc80/Hec1–Nuf2 and Spc24–Spc25 dimers. The dimers are joined at the ends of long alpha-helical coiled-coil domains extending from the N-terminal globular domains of Ndc80/Hec1–Nuf2 and the C-terminal globular domains of Spc24–Spc25. The N-terminal CH domains of Ndc80/Hec1–Nuf2 are involved with MT binding, as is the N-terminal unstructured amino acid tail of Ndc80/Hec1. A loop domain in the middle of the Ndc80/Hec1–Nuf2 alpha-helical coiled-coil domain makes Ndc80c flexible and provides a platform for binding MT-associated proteins^4^. The globular end of Spc24–Spc25 links the Ndc80c to kinetochores.

In human cells, the protein linkage between Spc24–Spc25 and chromatin within the inner kinetochore is only partly understood. CENP-C, CENP-T and the Mis12 complex are currently thought to play key roles (Fig. 1a,b). Inner kinetochore chromatin is defined by the presence of nucleosomes containing CENP-A, a modified Histone-H3, which functions as an epigenetic marker. CENP-C and CENP-T, which are part of the constitutive centromere-associated protein network (CCAN), bind CENP-A containing chromatin at their C-terminal domains^5^,^6^ and link Ndc80c to their N-terminal domains by different mechanisms (Fig. 1a,b)^7^. The Mis12 complex is part of the highly conserved KMN protein network that includes Knl1, Mis12 and Ndc80 complexes (Fig. 1b, label 1)^8^. The KMN network is part of the core attachment site for the plus ends of kMTs, and recruits the majority of outer kinetochore proteins including those of the spindle assembly checkpoint^2^,^9^,^10^,^11^,^12^.

Biochemical evidence has shown that the N terminus of CENP-C is capable of direct binding to the centromere–proximal end of the Mis12 complex and indirectly recruits Ndc80c by Spc24–Spc25 binding to the distal end of the Mis12 complex (Fig. 1a,b, label 2)^10^,^11^. Another site at the distal end of the Mis12 complex binds Knl1 (Fig. 1b, label 1). Only a minor fraction of CENP-C may be linked to the Mis12 complex (Fig. 1b, label 3)^13^,^14^,^15^.

CENP-T is known to recruit Ndc80c to kinetochores by directly binding to Spc24–Spc25. Biochemical studies have shown that the N-terminal domain (amino acid (aa) 1–100) of CENP-T can directly bind Spc24–Spc25 and prevent Spc24–Spc25 binding to the Mis12 complex (Fig. 1a,b, label 4)^16^,^17^. Super-resolution fluorescence microscopy has shown that the mean position of the N terminus of CENP-T co-localizes with Spc24–Spc25 at metaphase^13^. The above data predict that each CENP-T N terminus recruits an Ndc80c that is independent of the KMN network. Recent studies have shown that the N-terminal half of either CENP-T or CENP-C alone tethered to chromatin in high numbers by LacO–LacI (∼256) can establish a functional artificial kinetochore^18^. These artificial kinetochores lack CENP-A and the other CCAN proteins but contain all members of the KMN network and other outer kinetochore proteins like those of the spindle assembly checkpoint. Taken together, these findings indicate that CENP-T and CENP-C can both function independently as major recruiters of Ndc80c as well as the KMN network.

In this paper, we test quantitative predictions of the model described in Fig. 1b for how CENP-C, CENP-T and the Mis12 complex recruit Ndc80c to human kinetochores at metaphase. We obtained protein copy numbers per kinetochore for CENP-C, CENP-T, Mis12 complex members and Ndc80/Hec1 in normal HeLa cells. In addition, we made similar measurements for cells depleted of CENP-C but containing a chimera of the N-terminal half of CENP-T and DNA-binding domains of CENP-C to produce a ‘CENP-T' only linkage between the centromere and Ndc80c and/or KMN network. We also combined measurements of protein copy number with quantitative immunofluorescence assays of changes in protein numbers at kinetochores on selective protein depletion by RNA interference (RNAi).

We found that the picture in Fig. 1b must be modified since our measurements determined that there are on average 244 Ndc80c per human kinetochore, 215 CENP-C, 72 CENP-T and 151 KMN networks based on Mis12 measurements. Only 38% of CENP-C recruits a KMN network as predicted previously by super-resolution microscopy^13^, while each CENP-T recruits a KMN network in addition to the Ndc80c known to bind to the N-terminal end of CENP-T. In addition, the ‘CENP-T' only linkage to the outer kinetochore in the chimera cells produces functional kinetochores with the number of Ndc80/Hec1 and KMN networks predicted by the above stoichiometry. These data provide critical evidence for understanding the mechanical linkages between centromeric chromatin and kMT attachment sites at human kinetochores.

## Results

### Human kinetochore protein copy numbers

**Figure 1:**
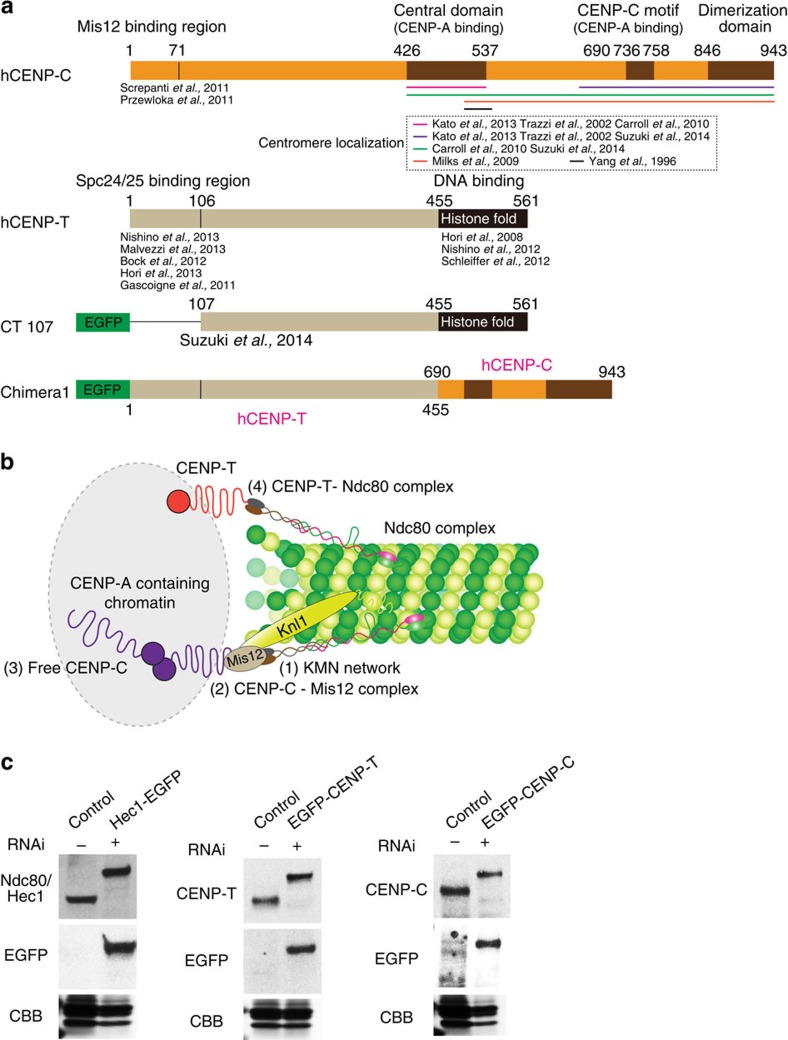
CENP-C and CENP-T are inner kinetochore proteins proposed to be primarily responsible for recruiting Ndc80c to kinetochores. (**a**) Schematic depiction of the domain organization of human CENP-C, CENP-T, CT107 (CENP-T 107–561 aa) and chimera1, which is a hybrid protein with CENP-T (1–455 aa) and CENP-C (690–943 aa). (**b**) Current thinking about CENP-C- and CENP-T-dependent linkages to Ndc80c as described in the text. (**c**) In our studies, the expression levels of EGFP fusion proteins in stably expressed cells are nearly identical to their endogenous proteins. Western blots for comparing the level of EGFP fusion protein in HeLa cell lines compared with wild-type (control) levels (top), Coomassie brilliant blue (CBB) staining of a loading control protein (bottom) and anti-GFP staining to confirm EGFP band (middle). Hec1–EGFP stable cells are (right), EGFP–CENP-T stable cells are (middle) and EGFP–CENP-C stable cells are (left). Endogenous proteins were depleted by RNAi in cells expressing an EGFP fusion protein.

We established clonal HeLa cells stably expressing enhanced green fluorescent protein (EGFP) fusion proteins for Ndc80/Hec1, CENP-T, CENP-C and three of the protein components of the Mis12 complex (Mis12, Dsn1 and Nnf1). Each EGFP fusion was expressed near the level of the endogenous protein following removal of the endogenous protein by RNAi (Fig. 1c; Table 1; Supplementary Fig. 1); the RNAi penetrance was sufficient to reduce the target protein levels to below the detection limit by both western blot (Fig. 1c) and immunofluorescence (Supplementary Fig. 2a). We confirmed by quantitative immunofluorescence that Ndc80/Hec1 localization at metaphase kinetochores and the mitotic index was comparable to control cells in all cell lines where EGFP fusions replaced the endogenous proteins (Table 2; Supplementary Fig. 2b,c). These results suggest that the EGFP fusions are able to functionally substitute for the endogenous proteins. All experiments using fluorescent protein fusion cells were performed with depletion of endogenous protein by RNAi unless otherwise noted.

**Figure 2:**
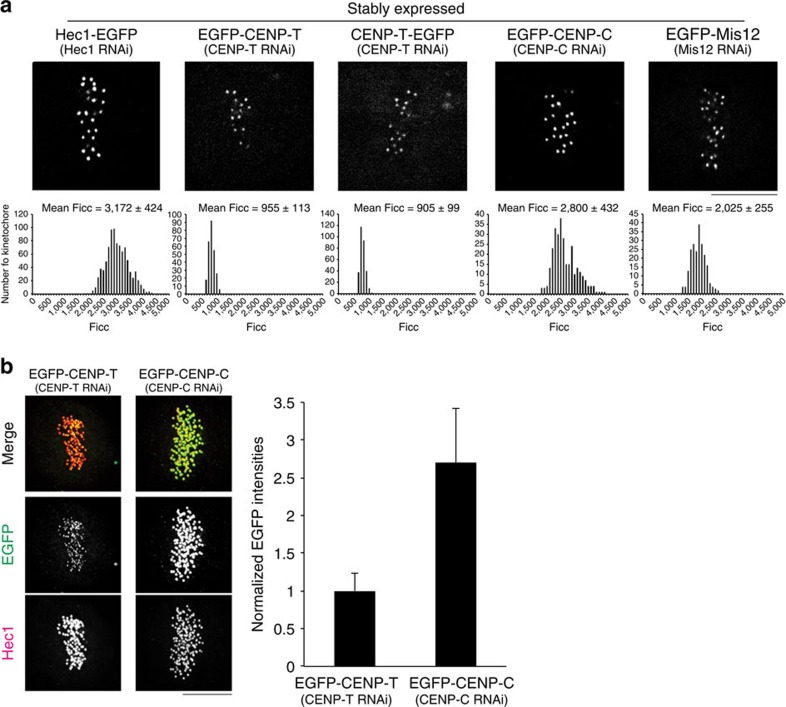
Summary of mean values of measured protein copy numbers per kinetochore at metaphase in control cells. (**a**) Mean values for Ficc (integrated kinetochore fluorescence minus background and corrected for kinetochore depth beneath coverslip and photobleaching) for EGFP at metaphase kinetochores in each cell line stably expressing an EGFP fusion protein (Summary of protein copy number values in Table 2). (**b**) Example of two-colour immunofluorescence (left) and EGFP kinetochore fluorescence measurements (right, *n*>150 kinetochores/>4 cells, See Methods) for EGFP–CENP-T and EGFP–CENP-C in stably expressing cells. All experiments including live cell imaging and immunofluorescence using cells expressing an EGFP fusion protein were depleted of endogenous protein by RNAi. Kinetochore intensities were normalized relative to EGFP–CENP-T intensities (**b**). Error bars are s.d. from the means. Scale bar, 5 μm.

We next measured protein copy numbers at metaphase kinetochores using a method described previously^19^,^20^. In brief, we obtained average kinetochore fluorescence intensities for in-focus kinetochores in live cells corrected for background intensity, depth beneath the coverslip and photobleaching (Ficc; Fig. 2a; Table 2; Supplementary Figs 3 and 4; see the Methods section). The average value for Ndc80/Hec1–EGFP was 3,172±424 (±s.d.) counts. This mean number corresponds to 244.0 molecules per kinetochore, based on 13 counts per EGFP measured *in vitro* at pH=7.1 (see the Methods section) and 14.3 molecules per kMT based on 17.1±0.6 kMTs per HeLa cell kinetochore as quantified by electron microscopy^21^. The value for EGFP–CENP-C was 2,800±432. The mean is 88% of the Ndc80/Hec1–EGFP fluorescence, and corresponds to 215.4 CENP-C molecules per kinetochore and 12.6 molecules per kMT (Table 2). For comparison, the value for CENP-T fused to EGFP was 931±109 (average of 3 different EGFP fusions), which is only 29% of the Ndc80/Hec1–EGFP intensity, and the mean corresponds to 71.6 molecules per kinetochore and 4.2 molecules per kMT (Table 2). To verify the above results, we performed quantitative immunofluorescence measurements with GFP antibodies. The results confirmed that EGFP–CENP-C intensities at kinetochores are >2.5 times EGFP–CENP-T intensities (Fig. 2b). The mean protein copy number measured for the Mis12 complex was 151.1 per kinetochore and 8.8 per kMT. These values are based on the average for the numbers measured for EGFP fusions to Mis12, Dsn1 and Nnf1 (Table 2), whose individual values were nearly identical as expected from biochemical data showing that the Mis12 complex contains one molecule of each^8^ (Table 2). We conclude that there are about 244 Ndc80/Hec1, 215 CENP-C, 72 CENP-T and 151 Mis12 complex per HeLa cell kinetochore on average at metaphase. These numbers indicate that the mean number of KMN networks per kinetochore is 151 because biochemical data have established for purified KMN network *in vitro* a 1:1:1 relationship between Mis12 complex, Ndc80c and Knl1 (not measured in this paper)^8^,^9^.

### Mis12 and CENP-T account for >90% of Ndc80c

The current hypothesis from biochemical data *in vitro* is that one CENP-T directly binds one Ndc80c (Fig. 1a,b), one Mis12 complex directly binds one Ndc80c (Fig. 1b), and CENP-T and Mis12 complex direct binding to the Ndc80c is mutually exclusive^16^,^17^,^22^,^23^. This hypothesis predicts that the number for Ndc80c=the number of CENP-T plus the number of Mis12 complexes on average per kinetochore. The sum of the means for CENP-T and Mis12 (Table 2) is 223±23 (calculation: 72+151±√(8.4^2^+21^2^)). This sum is 9% less than the measured value for Ndc80/Hec1 (244±32) providing strong support that the above hypothesis, which is based on *in vitro* biochemical data, is substantially correct *in vivo*. This conclusion is strengthened by the very low variation in mean protein counts per kinetochore measured for CENP-T for the three different cell lines expressing EGFP fusions and for the four different cell lines expressing EGFP fusions to various members of the Mis12 complex (Table 2). Immunofluorescence measurements for kinetochore Ndc80/Hec1 for these cell lines were also very similar to the value obtained for the Hec1–EGFP cell line (Table 2). A question remains as to whether the difference in the means, 244−223=21, represents another unknown linker protein at kinetochores responsible for binding these 21 Ndc80 complexes. It is likely that the mean count difference of 21 is just due to noise in the measurements since the protein counts were obtained from different cell lines where the mean level of Ndc80 at kinetochores differed by about s.d.=±15% based on quantitative immunofluorescence (Table 2).

### CENP-T and CENP-C contributions

**Figure 3:**
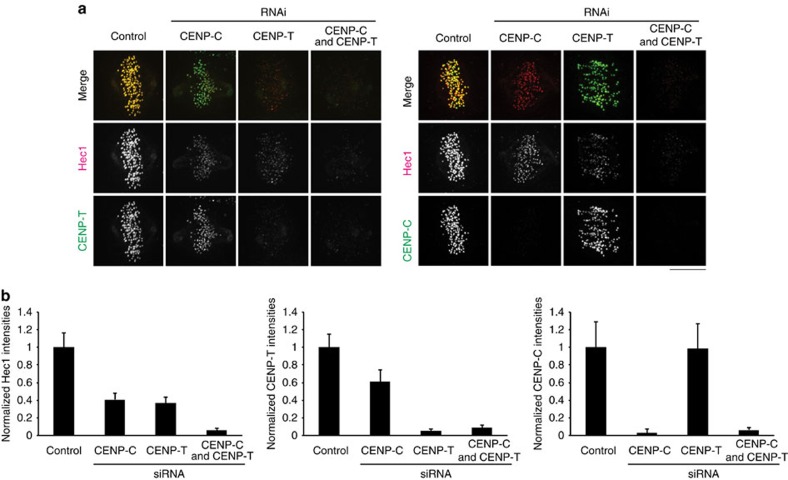
Stoichiometry of Ndc80/Hec1 recruitment to kinetochores by CENP-T and CENP-C in metaphase cells. (**a**) Typical images of two-colour immunofluorescence of anti-Hec1 and anti-CENP-T (left), anti-Hec1 and anti-CENP-C (right) in control and CENP-T RNAi-, CENP-C RNAi- and CENP-T/-C RNAi-treated cells. (**b**) Mean kinetochore intensities of Hec1, CENP-T and CENP-C normalized by corresponding control values in each condition of (**a**). *n*>200 kinetochores/>5 cells, See Methods. Error bars are s.d. from the mean. Scale bar, 5 μm.

To determine the contributions of CENP-T and CENP-C to Ndc80c recruitment, we measured the kinetochore fluorescence intensity of all the three proteins in CENP-C, CENP-T and CENP-C/CENP-T-depleted cells by immunofluorescence in fixed late prometaphase or metaphase cells (Fig. 3a,b; Table 3a). Depletion of CENP-C reduced Ndc80/Hec1 and CENP-T to 41% and 61% of control, respectively. In contrast, depletion of CENP-T reduced Ndc80/Hec1 to 37% of control without a significant reduction of CENP-C. This suggested that CENP-C is involved in recruiting CENP-T to kinetochores at metaphase, but at a lower contribution than reported for interphase^24^. We found that double depletion of CENP-C and CENP-T reduced Ndc80/Hec1 to less than 5% of control in prometaphase (Table 3a), confirming that these two proteins coordinately recruit Ndc80/Hec1 to metaphase kinetochores^25^,^26^.

We next used the results of our kinetochore protein copy number measurements in metaphase control cells (Table 2) to calculate the number of CENP-T, CENP-C and Ndc80/Hec1 molecules remaining at kinetochores for each RNAi condition. The results shown in Table 3a indicate that CENP-T recruitment of Ndc80/Hec1 is largely independent of CENP-C.

RNAi depletion of CENP-T from 72 to 4 molecules on average per kinetochore caused a drop in Ndc80/Hec1 from 244 to 90 molecules, a loss of 154 molecules. This number is more than twice the number of CENP-T molecules removed by RNAi, 68. As depletion of CENP-T does not affect CENP-C levels, these measurements indicate that each CENP-T molecule recruits ∼2 Ndc80/Hec1 molecules to the kinetochore.

For CENP-C, the situation is more complex. RNAi depletion of CENP-C from 215 to 6 molecules on average per kinetochore reduces Ndc80/Hec1 from 244 to 100 molecules. Although this number is similar to the loss of 209 CENP-C molecules caused by the RNAi, the effect of the 39% reduction in CENP-T following CENP-C depletion needs to be accounted for. Taking the reduction of CENP-T into consideration, the number of CENP-C molecules that directly recruit Ndc80/Hec1 must be significantly less than the 144 predicted by the model in Fig. 1b.

To better quantify the number of Ndc80/Hec1 molecules recruited by CENP-T and CENP-C as well as the fraction of CENP-C involved in recruiting Ndc80/Hec1, we solved the following equations, where NT is the mean total number of Ndc80/Hec1 molecules at the kinetochore and TN and CN are the mean numbers for CENP-T and CENP-C recruited Ndc80/Hec1 molecules, respectively:


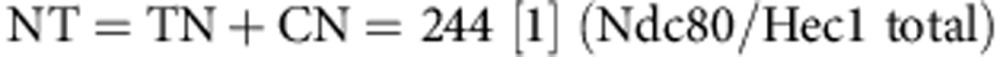



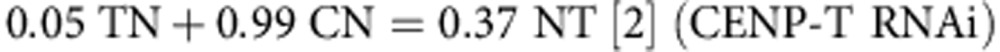



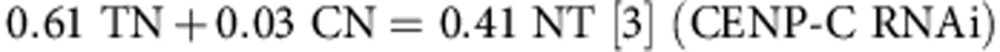


The solution to these equations (Supplementary Note 1) yields ∼160–161 and ∼83–84 Ndc80/Hec1 recruited by CENP-T and CENP-C, respectively. Since the mean total kinetochore copy number for CENP-T and CENP-C is 72 and 215, respectively, this analysis predicts that each CENP-T recruits on average 2.2 Ndc80 complexes and only 39% of the kinetochore-localized CENP-C recruits an Ndc80 complex, assuming 1 Ndc80/Hec1 is linked to CENP-C through the KMN network. Reducing the value of NT to 223, the measured sum of mean protein counts for CENP-T and Mis12 direct linkers to the Ndc80c (Table 2), yields 2.0 Ndc80c per CENP-T and 35–36% of CENP-C that recruits an Ndc80 complex (Supplementary Note 1). If 35–39% of CENP-C recruits only ∼76–84 Ndc80/Hec1 to the kinetochore as part of the KMN network, then another significant linkage to the KMN network besides CENP-C must exist since the mean protein copy number we measured for Mis12 per kinetochore is almost twice as big, ∼151 (Table 2).

How well the above data reflect the amount of endogenous protein at metaphase kinetochores depends on the mean cellular concentrations of the EGFP fusion protein relative to endogenous and whether kinetochore binding sites are normally saturated at endogenous protein concentration. The data in Table 1 indicate that the concentrations of EGFP fusions are close to endogenous values, but not exact. To test whether this is critical, we examined how the amounts of metaphase kinetochore Ndc80/Hec1, CENP-T and CENP-C depend on protein overexpression. We found the amount of Ndc80/Hec1 at metaphase kinetochores was not dependent on overexpression of either EGFP–CENP-T or EGFP–CENP-C (Supplementary Fig. 6a). This result indicates that endogenous levels of Ndc80/Hec1 are limited by a finite number of binding sites. This conclusion is also supported by experiments where we transiently expressed Hec1–mCherry in cells stably expressing Hec1–EGFP. We found that Hec1–EGFP intensities at kinetochores decreased as Hec1–mCherry intensity increased (Supplementary Fig. 6b). A similar result was found for cells stably expressing Hec1–mCherry or Hec1–tdTomato and challenged with overexpression of Hec1–EGFP (Supplementary Fig. 6b). Next, to test whether the amount of CENP-T or CENP-C at metaphase kinetochores is normally limited by a finite number of binding sites, we performed similar experiments using EGFP–CENP-T or EGFP–CENP-C stably expressed cells. We found that the amount of EGFP–CENP-T or EGFP–CENP-C at kinetochores decreased as the corresponding amounts of mCherry–CENP-T or mCherry–CENP-C at kinetochores increased with protein overexpression (Supplementary Fig. 6c). The above results strongly suggest that a limited number of binding sites for Ndc80/Hec1, CENP-T and CENP-C are saturated at metaphase kinetochores independent of protein overexpression at levels near endogenous values.

### CENP-T(107–455 aa) recruits a KMN network

**Figure 4:**
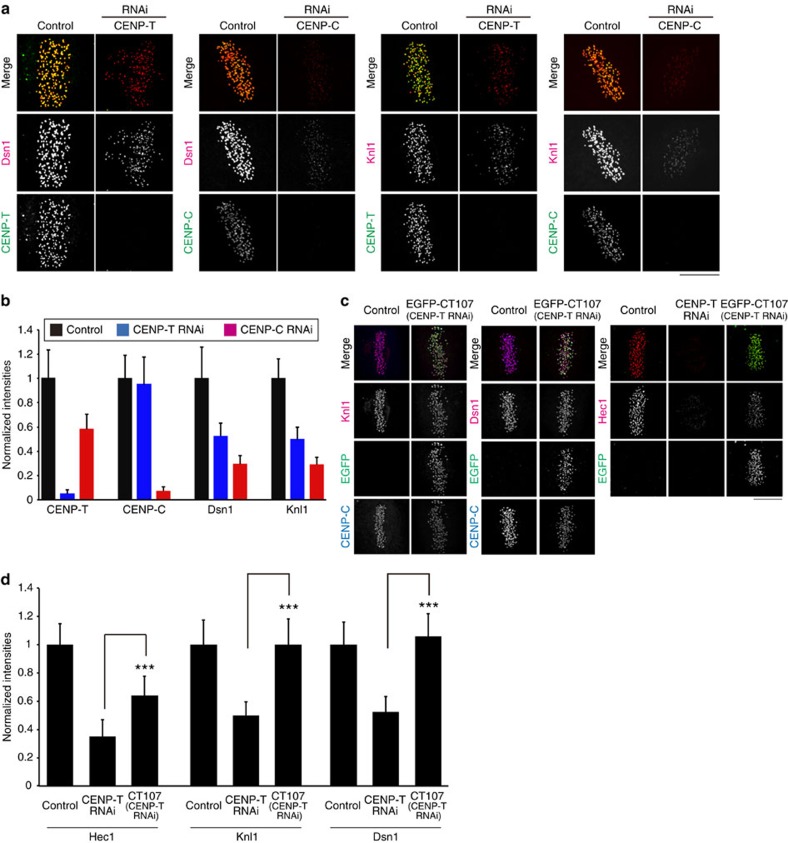
Stoichiometry of Mis12 complex and Knl1 recruitment to kinetochores by CENP-T and CENP-C in metaphase cells. (**a**) Typical two-colour immunofluorescence images of kinetochores labelled with anti-Dsn1 or anti-Knl1 and anti-CENP-T or anti-CENP-C in control, and CENP-T RNAi- and CENP-C RNAi-treated cells. (**b**) Mean values of kinetochore intensities normalized by control values for CENP-T, CENP-C, Dsn1 and Knl1. *n*>200 kinetochores/>5 cells (see the Methods section). (**c**,**d**) Examples of three-colour immunofluorescence of kinetochore Hec1, Dsn1 and Knl1 intensities in control cells, cells treated with CENP-T RNAi, or EGFP–CT107, stably expressed cells after CENP-T RNAi (**c**). Plots of mean kinetochore intensity of Hec1 (*n*>330 kinetochores/>9 cells, (see the Methods section), Knl1 (*n*>400 kinetochores/>11 cells) and Dsn1 (*n*>340 kinetochores/>9 cells) normalized by control values for the cells (**d**). Error bars are s.d. from the means. Scale bar, 5 μm. ***P<0.01 (*t*-test).

Biochemical evidence indicates that the N terminus of CENP-T recruits a single Ndc80c by binding to Spc24/Spc25, but the data in Supplementary Note 1 indicate that CENP-T at metaphase kinetochores is recruiting about two Ndc80c. Current structural studies^16^,^17^ show that the N-terminal end of CENP-T directly binds Spc24/Spc25, and this CENP-T–Spc24/Spc25 binding competes with Mis12–Spc24/Spc25 binding based on gel filtration experiments. On the other hand, recent studies^18^,^25^ indicate that a site within CENP-T between amino acids 1–530 (Chicken) or whole CENP-T (human) might be capable of recruiting the Mis12 and Knl1 members of the KMN network. This suggests that the additional Ndc80c recruited by CENP-T is indirectly recruited as part of a KMN network. To test this hypothesis, we quantified by immunofluorescence the percentage relative to control metaphase kinetochores of Dsn1 (a Mis12 complex component) and Knl1 remaining after CENP-T and CENP-C RNAi (Fig. 4a,b, Table 3b). CENP-T RNAi reduced both about 50%, while CENP-C RNAi reduced both by about 70% (Table 3b). The solutions to simultaneous equations 1–3 (Dsn1), 4–6 (Knl1) in Supplementary Note 2 yielded values indicating that about 44–48% of Mis12 complex and 44–50% of Knl1 depend on CENP-T for their recruitment to metaphase kinetochores. This result predicts that in addition to directly recruiting Ndc80c by binding to Spc24/Spc25, CENP-T indirectly recruits an additional Ndc80c by recruiting a KMN network. In addition, the calculations in Supplementary Note 2 yielded ∼37–40% of total CENP-C bound to Mis12, a fraction nearly identical to the analysis in Supplementary Note 1.

To test whether KMN network recruitment by CENP-T is independent of the N-terminal end of CENP-T that directly binds the Spc24/25 globular domains of Ndc80c^16^,^17^, we established a cell line stably expressing RNAi resistant CT107 (Fig. 1a). CT107 lacks the first 106 aa of CENP-T (described in detail in ref. 13). These cells stably expressing EGFP–CT107 maintained around 60% of Ndc80/Hec1 compared with controls (Fig. 4c,d). This percentage is predicted by previous studies in DT40 chicken cells^18^. In contrast, both Knl1 and Dsn1 had the same level at kinetochores as controls (Fig. 4c,d). These results show that CENP-T recruits a KMN network independent of the N-terminal end of CENP-T that directly binds to Spc24/Spc25.

### Chimera1 cells without CENP-C have functional kinetochores

**Figure 5:**
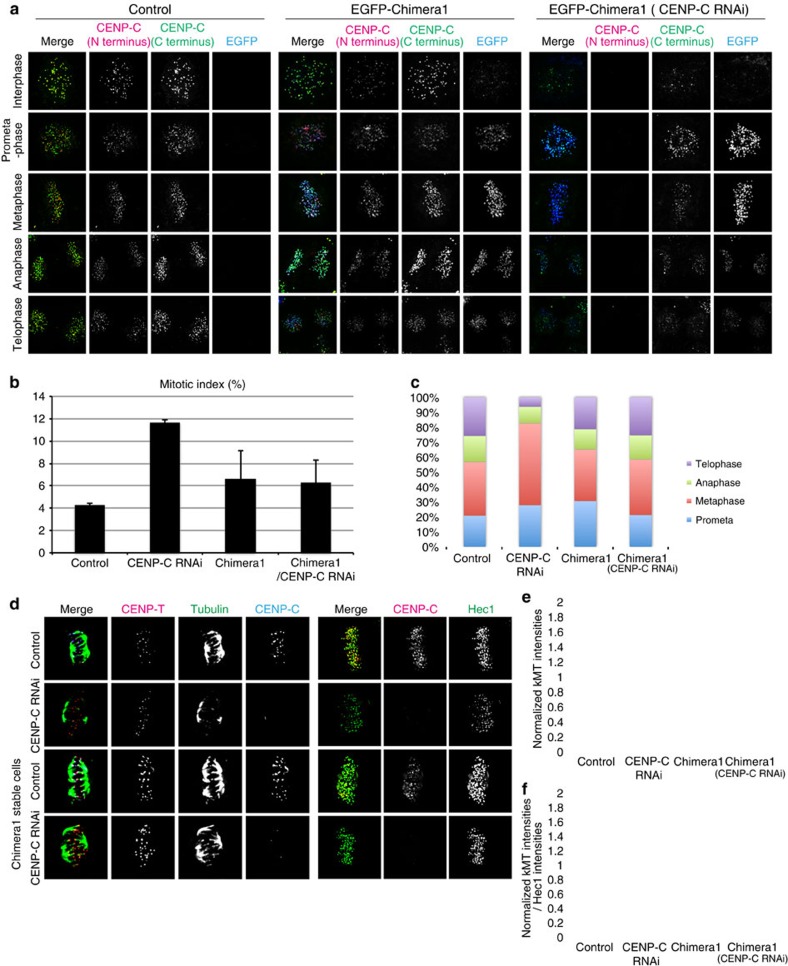
Mitotic kinetochores with only CENP-T linkage are sufficient for chromosome segregation. (**a**) Representative immunofluorescence images of kinetochores stained with anti-CENP-C (N terminus or C terminus, which only recognized endogenous CENP-C) and anti-GFP during cell cycle in control, EGFP–chimera1 and EGFP–chimera1 cells with treated with CENP-C RNAi. (**b**) Mitotic index for each condition in **a** and CENP-C RNAi cells. (**c**) The ratio of prometaphase, metaphase, anaphase and telophase within mitosis for each condition in **b** showing that the hybrid EGFP–chimera1 protein rescued CENP-C-depletion phenotype. (**d**) Representative immunofluorescence images of kinetochores in cells with cold-stable kMTs at metaphase stained with antibodies to CENP-T, tubulin and CENP-C (left) and antibodies to CENP-C and Hec1 (right) for each condition in **b**. (**e**) Mean cold-stable kMT intensities (*n*>60 kMTs) in each condition of **b** normalized by control value. (**f**) Values for cold-stable kMT intensities in **e** normalized by Hec1 intensities in each condition. Error bars are s.d. from the means. Scale bar, 5 μm.

The above analysis shows that there are two pools of Ndc80c, one recruited by ∼38% of CENP-C and the other by CENP-T. It is possible that the two pools have functionally distinct properties, which are important for chromosome segregation, or if not functionally distinct, the sum of their numbers is important. To address this issue, we generated a stable cell line expressing chimera1 protein in which CENP-T(1–455) was fused to CENP-C(690–943) (Fig. 1a). CENP-T(1–455) lacks the CENP-T DNA-binding domain, but contains the motif that directly binds to Spc24/Spc25 of Ndc80c^16^,^17^ as well as an unknown domain involved in recruiting the KMN network to kinetochores. Conversely, CENP-C(690–943) lacks the N-terminal binding region for the Mis12 complex that recruits the KMN network, but retains partial centromeric chromatin-binding domains and the dimerization domain of CENP-C (Fig. 1a)^5^. Chimera1 localized at kinetochores throughout the cell cycle, similar to CENP-C (Fig. 5a). Cells stably expressing chimera1 exhibited normal mitotic progression when endogenous CENP-C was depleted by RNAi (Fig. 5a–c). Thus, chimera1 supports chromosome segregation in the absence of CENP-C.

As a further test for the ability of the kinetochore to function normally in chimera1 stably expressed cells after CENP-C RNAi, we assayed the cold stability of kMTs at metaphase. A previous study found that the amount of cold-stable kMTs is proportional to the amount of kinetochore Ndc80/Hec1 (ref. 27). We measured kMT intensities in control, CENP-C-depleted cells, and chimera1 cells with and without CENP-C depletion and cold treatment before fixation (Fig. 5d,e). We found that kMT intensities were around 60% reduced in CENP-C-depleted cells after cold treatment before fixation compared with that of the control. However, cold-stable kMT intensities were exhibited by chimera1 stably expressed cells at control levels even when these cells did not have CENP-C at kinetochores (Fig. 5e). As predicted in a previous study^27^, cold-stable kMT intensities normalized by Ndc80/Hec1 kinetochore intensities in each condition were constant (Fig. 5f). Thus, stability of kMT anchorage at kinetochores depends on the amount of Ndc80/Hec1 molecules, independent of recruitment by CENP-T or CENP-C.

**Figure 6:**
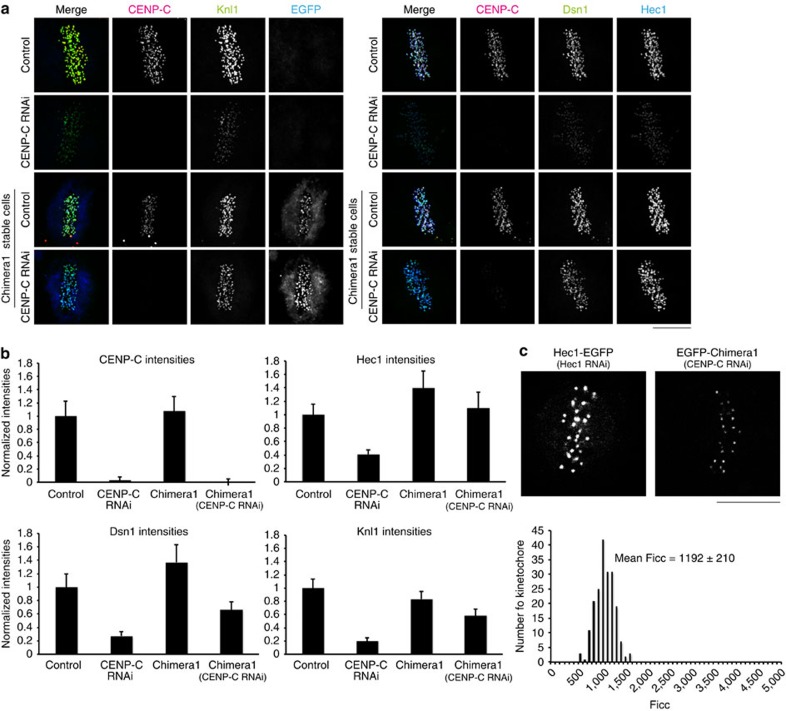
CENP-T(1–455) recruits a KMN network independent of CENP-C. (**a**) Representative immunofluorescence images of antibodies to CENP-C, GFP, Knl1, Dsn1 and Hec1 in control, CENP-C RNAi-treated cells, EGFP–chimera1 or EGFP–chimera1 cells treated with CENP-C RNAi. (**b**) Mean values for immunofluorescence intensities at kinetochores for CENP-C (*n*>120 kinetochores/>3 cells; see the Methods section), Hec1 (*n*>200 kinetochores/>5 cells), Dsn1 (*n*>150 kinetochores/>4 cells) and Knl1 (*n*>150 kinetochores/>4 cells) normalized by control values for each condition in **a**. (**c**) Example live-cell images of Hec1–EGFP cells or EGFP–chimera1 cells (top). The histogram of Ficc measured for EGFP–chimera1 after CENP-C depletion (bottom). Scale bar, 5 μm. Note, a chicken GFP antibody was needed to label EGFP–chimera1 in **a**. The non-specific cytosol staining was not exhibited by the rabbit GFP antibody used in other assays (for example, Supplementary Fig. 7c).

The mean number per kinetochore of Ndc80/Hec1 for the chimera1 cells following CENP-C RNAi (Fig. 6a,b) was 110% of the value for control cells measured by quantitative immunofluorescence. This result yields 268 Ndc80c on average per kinetochore based on the protein copy number for control kinetochores (Table 3c) and indicates that the N-terminal CENP-C motif that recruits Ndc80c indirectly via association with a KMN network is not needed to achieve a normal amount of Ndc80c per kinetochore in the chimera1 cells.

If each CENP-T recruits ∼2 Ndc80c in the chimera1 cells depleted of CENP-C, then we expected that the protein copy number for the chimera1 protein should be similar to but larger than the number for CENP-T, since the total number of Ndc80/Hec1 for the chimera1 cells is 110% of the value for control cells. To address this question, we measured integrated kinetochore fluorescence of EGFP–chimera1 in living cells following depletion of CENP-C (Fig. 6c; Table 2). The Ficc of chimera1 was 1192±210 counts, which corresponds to 92 molecules per kinetochore and 5 molecules per kMT on average. These values are ∼130% of the corresponding values for CENP-T in control cells (72 and 4.2, respectively) indicating that the great majority of chimera1 at kinetochores is recruiting Ndc80c in the same way as CENP-T itself. These numbers are also less than half the values measured for CENP-C in control cells (215 and 12.6, respectively, Table 2). This is likely because chimera1 lacks one of the DNA-binding domains of CENP-C (Fig. 1a), whose deletion from CENP-C has been shown to reduce the amount of CENP-C at kinetochores^5^.

To confirm this finding, we established a cell line stably expressing chimera2, which contains CENP-T(1–455) domain and the additional DNA-binding domain of CENP-C missing in chimera1 (Supplementary Fig. 7a). As predicted by a previous study^5^, chimera2 exhibited twice the concentration of chimera1 at kinetochores after endogenous CENP-C depletion. The level for chimera2 was very close to the level of CENP-C–EGFP in control cells (Supplementary Fig. 7b,c). Although chimera2 was twice the level of chimera1 at kinetochores in CENP-C-depleted cells, the mean level of Ndc80/Hec1 was nearly identical to the level in control cells. Why chimera2 is unable to recruit more Ndc80/Hec1 is unknown and the answers are likely complex since only 38% of CENP-C at control kinetochores recruits a KMN network (Supplementary Notes 1 and 2). As a result, in this paper we focus on chimera1.

### Chimera1 recruits 1 Ndc80c and 1 KMN network

As there is almost no CENP-C in the chimera1 cells depleted of CENP-C by RNAi, most of the 268 Ndc80c on average per kinetochore (Table 3c) must be recruited by endogenous CENP-T and the 92 EGFP–chimera1 molecules per kinetochore that are present on average after endogenous CENP-C depletion (Table 3c). Note that the analysis of CENP-C-depleted control cells in Fig. 6 was performed independent of the experiments that yielded the data in Fig. 4, but the numbers from the two different experiments were very similar to each other. We assumed the number of CENP-T remaining after depletion of CENP-C in control cells, 38, for the number of CENP-T remaining after depletion of CENP-C in the chimera1-expressing cells. This value accounts for the immunofluorescence data showing that CENP-T intensities in chimera1 cells depleted of CENP-C was 1.8 times larger than control because CENP-T antibodies recognized both endogenous CENP-T and the CENP-T(1–455) domain of chimera1 (Supplementary Fig. 7d). In addition, CENP-T intensity in chimera1 depleted of both CENP-T and CENP-C, which represents only the CENP-T part of chimera1, was 1.2 times brighter than control (Supplementary Fig. 7d). This value corresponds to the protein copy number of EGFP–chimera1 (Table 3c). Using the assumed value for remaining endogenous CENP-T in chimera1 cells depleted of CENP-C, each CENP-T(1–455) domain contributed 268/(38+92)=268/130=∼2 Ndc80c (Supplementary Note 3). This result is very similar to the measured number of 2.0–2.2 obtained for CENP-T in control cells in the presence of normal CENP-C (Supplementary Note 1) and strongly supports our hypothesis that CENP-T recruits 2 Ndc80c.

When we depleted endogenous CENP-T as well as CENP-C by RNAi in chimera1-expressing cells, Ndc80/Hec1 was reduced by about 40% (Supplementary Fig. 7d). These data strongly support both our protein counting results and stoichiometry measurements of Ndc80/Hec1 recruitment to kinetochores (Supplementary Notes 1 and 3). In addition, a 40% reduction in the Ndc80/Hec1 is significant as it slows mitotic progression, by delaying chromosome alignment at the metaphase plate^27^.

We next tested whether the CENP-T(1–455) domain in chimera1 recruits the KMN network to kinetochores in addition to recruiting an Ndc80c by binding directly to Spc24/Spc25. As we did for Ndc80/Hec1, we used quantitative immunofluorescence to measure the change in level of the other members of the KMN network after CENP-C RNAi in control cells and chimera1-expressing cells. In control cells, non-Ndc80c members of the KMN network (Dsn1 and Knl1) were all reduced to 20% of control levels by RNAi of CENP-C (Table 3), results similar to those reported in a previous study^25^. If this 20% level of KMN network at kinetochores is solely contributed by recruitment of 38 CENP-T per kinetochore as measured for CENP-C-depleted control cells (Table 3), then the addition of 92 chimera1 per kinetochore should increase the percentage to (38+92) × (20)/38=68% of control if the CENP-T(107–455) domain is solely responsible for recruiting the KMN network. The levels of the non-Ndc80c KMN network proteins in chimera1 cells depleted of CENP-C were 58–66% of control values (Table 3c) demonstrating recruitment of KMN network proteins by CENP-T(1–455) domain in chimera1. In addition, the kinetochore level of these KMN components in chimera1 cells depleted of CENP-C confirmed that CENP-T(1–455) domain of CENP-T is able to recruit one KMN network independent of the N terminus of CENP-C (Supplementary Note 3).

## Discussion

**Figure 7:**
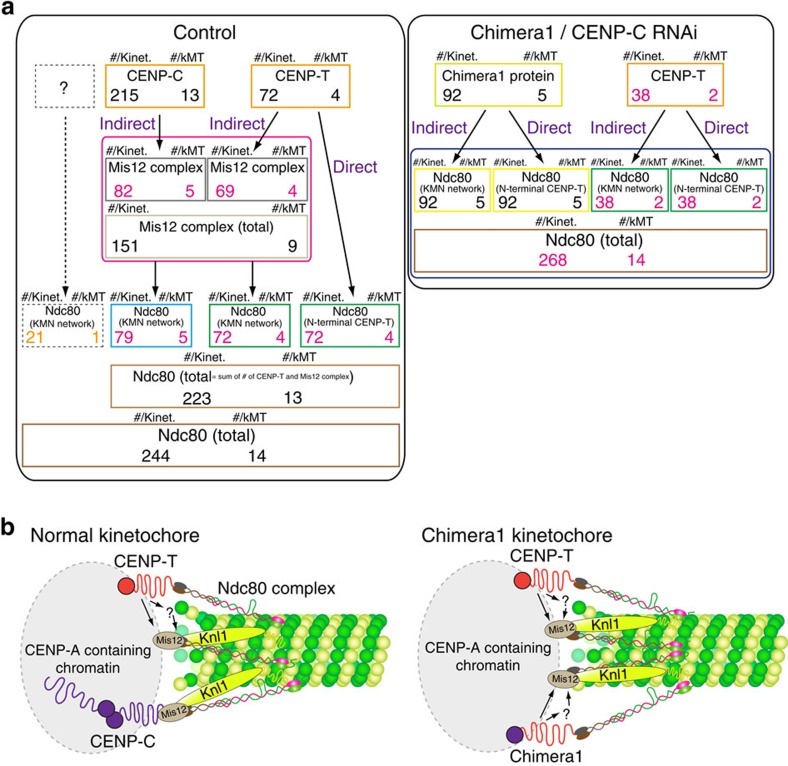
A quantitative summary of how CENP-T and CENP-C (controls cells) or CENP-T and chimera1 recruit Ndc80/Hec1 to kinetochores. (**a**) Summaries of the mean protein copy numbers per kinetochore or per kMT and the mean numbers of Ndc80/Hec1 recruited directly or indirectly as part of the KMN. (**b**) Updated diagram from Table 2 based on summary data in **a** for CENP-C- and CENP-T-dependent linkages to Ndc80c and the KMN network at kMT plus ends for normal kinetochores and for chimera1 kinetochores in cells depleted of CENP-C. At kinetochores in control cells, 1 CENP-T recruits ∼2 Ndc80c; one is independent of the KMN network and another is part of the KMN network. Only ∼38% of CENP-C recruits an Ndc80c and it is part of a KMN network. For kinetochores in the chimera1 cells, both CENP-T and the chimera1 protein each recruit two Ndc80c; one directly and the other indirectly by recruitment of a KMN network. In **a**, the protein copy numbers for CENP-C, CENP-T, Mis12 complex and Ndc80/Hec1 in control cells and in chimera1 cells depleted of CENP-C are derived from Table 2, and Supplementary Notes 1 and 3. The black copy numbers were measured from EGFP fluorescence, the red numbers from quantitative immunofluorescence and the orange numbers from the Ndc80/Hec1 contributed by the sum of CENP-T and Mis12 measured numbers. In the chimera1 cells depleted of CENP-C, we had to assume the same value for CENP-T as we measured for control cells after CENP-C depletion (Table 3). We used a mean value of 17.1 kMTs per kinetochore for control^21^, and a mean value of 18.8 kMTs per kinetochore for chimera1 cells depleted of CENP-C, because kMT intensities under cold treatment in chimera1 cells depleted of CENP-C were 10% higher than control (Fig. 5e).

The tables in Fig. 7a summarize our results for protein copy numbers at human kinetochores and the stoichiometry of Ndc80/Hec1 recruitment at metaphase in control human cells (Fig. 7a, left) and chimera1 cells after CENP-C depletion (Fig. 7a, right). The protein copy numbers in Fig. 7a are derived from the measured protein copy numbers in Table 2 and Supplementary Notes 1 and 3. The analysis for control cells determined that each CENP-T recruited 2.0–2.2 Ndc80/Hec1 (Fig. 7a, left). The analysis of chimera1 cells determined that each CENP-T(1–455) domain recruited 2.0 Ndc80/Hec1 (Fig. 7a, right). On the basis of the accuracy of our data, 2 is probably correct for control cells based on our measurement accuracy and ∼21 kinetochore Ndc80/Hec1 may be recruited by an unknown mechanism (Fig. 7a, left).

The human kinetochore in control cells has on average 244 Ndc80/Hec1 molecules (Fig. 7a, left). The mean copy number of Ndc80/Hec1 proteins per kMT is well conserved: budding yeast (17.4±2.1, re-calibrated by 13 counts/EGFP from previous data^19^); chicken DT40 (18.1±3.2, re-calibrated by 13 counts/EGFP from previous data^20^); and human (14.3±1.9, Fig. 7a, left). The mean protein copy number for Ndc80/Hec1 at kinetochores of chimera1 cells depleted of CENP-C is slightly higher than controls, 268, with 15.6 per kMT based on fluorescence measurements showing a slightly higher number for cold-stable kMTs per kinetochore and for Ndc80/Hec1 (Fig. 7a, right). These cells progressed normally through mitosis indicating that it does not matter whether Ndc80 complex is recruited by CENP-T or CENP-C when the number of Ndc80/Hec1 per kMT is near normal.

Our results (Fig. 7a) show that CENP-T and CENP-C have very different stoichiometry for recruiting Ndc80c, although both are major recruiters of Ndc80c at vertebrate kinetochores^18^,^25^. Kinetochores in control cells have on average 215 CENP-C (13 CENP-C molecules per kMT) and 72 CENP-T molecules (4 CENP-T molecules per kMT) that recruit 151 Mis12 complexes (9 Mis12/Dsn1/Nnf1 molecules per kMT) and 244 Ndc80/Hec1 molecules (14 Ndc80/Hec1 molecules per kMT; Fig. 7a, left). Protein copy measurements for both control and Chimera1 cells indicate that CENP-T(1–455) recruits a KMN network in addition to a Ndc80c (Fig. 7a, green boxes). We also found that CENP-T(107–455) was capable of recruiting a KMN network, but not the Ndc80/Hec1 that depends on the N-terminal 100 aa binding to Spc24/Spc25 (Fig. 4c,d).

Taken together, the above data indicate that the CENP-T(1–455 aa) domain has a critical role for recruitment of a KMN network in addition to independently binding to an Ndc80c (Fig. 7a,b). This conclusion is also strongly supported by recent papers showing that either CENP-T or CENP-C alone can recruit the KMN network and establish artificial functional kinetochores using a LacO/LacI linkage to chromatin in human cells and chicken DT40 cells^18^,^25^. Also, our results are supported by a recent paper showing for chicken DT40 cells that a mutation in Spc25 (I156R), which prevents binding to the N terminus of CENP-T *in vitro*, causes partial loss of Ndc80c at metaphase kinetochores *in vivo*^17^. How the CENP-T(107–455) domain recruits KMN network proteins at human kinetochores at metaphase is not yet known. Current possibilities include binding a KMN network directly^18^,^25^,^28^ or indirectly through the CENP-H/-I complex^29^,^30^, whose recruitment depends strongly on CENP-T, but not on CENP-C^26^. Future studies are needed to resolve just how CENP-T recruits a KMN network.

Our data indicate that each of the 72 CENP-Ts at a normal metaphase kinetochore recruits about 2 Ndc80/Hec1, while only 39% (100 × 83/215) of 215 CENP-C within the inner kinetochore have their N-terminal domains linked to the KMN network in the outer kinetochore. These results are predicted by measurements of the mean positions of the N-terminal ends of CENP-T and CENP-C using a two-colour super-resolution fluorescence method^13^. The N terminus of CENP-T is localized close to the position of Spc24 globular domain indicating that the great majority of CENP-T N terminus is bound to the Ndc80c^13^. In contrast, the N terminus of CENP-C localized ∼30 nm interior from Mis12 complex^13^,^14^,^15^. These results are consistent with the majority of CENP-C being free of the Mis12 complex and using both its ends to strengthen the structural integrity of the inner kinetochore as proposed by a previous study^31^.

Vertebrate kinetochores normally need both CENP-C and CENP-T to build a functional kinetochore^13^,^32^. For example, in cells depleted of either CENP-C or CENP-T, the amount of Ndc80/Hec1 is reduced by >50% and cells are unable to progress through mitosis properly (Table 3). We found that kMT stability was reduced depending on the number of Ndc80/Hec1 molecules (Fig. 5d–f) as predicted by a previous study that depleted Ndc80/Hec1 by various amounts using RNAi^27^. However, we also showed that the stability of kMT assembly was independent of Ndc80/Hec1 recruitment by CENP-T or CENP-C providing a sufficient number of Ndc80c was recruited (Fig. 5d–f). This result explains why artificial kinetochores built from ∼256 LacI/LacO attached to either CENP-T or CENP-C alone are able to support chromosome segregation^18^,^25^.

We only have a partial answer for the question about why vertebrate kinetochores require both CENP-T and CENP-C, since both CENP-T and CENP-C can recruit KMN network independent of each other (Fig. 7a,b)^18^,^25^. It might be because CENP-C or CENP-T alone cannot recruit enough Ndc80c on the native kinetochore for stable assembly of kMTs.

It is also an interesting mystery why CENP-T is required for faithful chromosome segregation by vertebrate kinetochores, despite there being potentially enough numbers of CENP-C per kinetochore to recruit the number of Ndc80c normally recruited by CENP-T and CENP-C together. In budding yeast, Cnn1 (human CENP-T) has the conserved function for recruitment of Ndc80c, but it is not essential for chromosome segregation and it only localizes to kinetochores at anaphase^22^,^23^,^33^. In addition, CENP-T is not identified yet in *Drosophila*, where Ndc80c appears recruited only as part of the KMN network by CENP-C^10^. However, in vertebrates, both CENP-C and CENP-T have poorly understood functions beyond recruiting Ndc80c and KMN that involve the composition and structural integrity of CENP-A chromatin within the inner kinetochore^34^.

## Methods

### Cell culture and RNAi

HeLa cells were cultured in Dulbecco's modified Eagle's medium (Invitrogen) supplemented with 10% fetal bovine serum (Sigma), 100 U ml^−1^ penicillin and 100 mg ml^−1^ streptomycin at 37 °C in a humidified atmosphere with 5% CO_2_. RNAi experiments were conducted using LipofectamineRNAi MAX (Invitrogen) according to the manufacturer's instructions. Short interfering RNA (siRNA) transfections were performed with a total of 100 nM of siRNA duplex and siRNA sequences of CENP-T and CENP-C described previously^13^. For Ndc80/Hec1 RNAi (5′-GAUACUUGCACGGUUUACAGA-3′ and 5′-CCCUGGGUCGUGUCAGGAA-3′), Mis12 RNAi (5′-GCAAAAUAAGCCAAGAUGUCU-3′, GUAUCUAUGCCAAUUUGUUUU-3′) was used.

### Stable cell lines expressing fluorescent fusion proteins

Stable cell lines were established with: EGFP–CENP-T, CENP-T–EGFP, EGFP–CENP-C, Hec1–EGFP, Hec1–mCherry, Hec1–tdTomato, EGFP–Mis12, Dsn1–EGFP, EGFP–Dsn1, EGFP–Nnf1, EGFP–CT107, EGFP–chimera1 (CENP-T (1–455 aa) fused with CENP-C (690–943 aa)) and EGFP–chimera2 (CENP-T (1–455 aa) fused with CENP-C (426–943 aa); see Supplementary Fig. 4c for detailed parameters). All EGFP-fused proteins were driven by CMV (human cytomegalovirus immediate early) promoter. All plasmids were linearized by restriction enzymes, and then purified plasmids were transfected using Effectene regents (Qiagen) according to the manufacturer's instructions. After transfection, Neomycin (Gibco) or Puromycin (Gibco) was added. We collected >20 individual positive colonies from each transfection then we tested expression level by western blot and immunofluorescence. We selected cells expressing EGFP fusion protein at a similar level to the endogenous protein as measured by western blot and immunofluorescence. We tested whether Hec1 level at kinetochores in metaphase was similar to control cells in all EGFP fusion stably expressed cell lines (Supplementary Fig. 2c). All cells were clonal cell lines. The cell line stably expressing EGFP–LAP–CENP-T was obtained from Dr I. M. Cheeseman (MIT, Whitehead).

### Immunoblotting

Cells were suspended in lysis buffer (50 mM Na Phosphate pH 8.0, 30 mM NaCl, 0.1% NP40, 5 mM β-mercaptoethanol, protease inhibitors (Roche) and phosphatase inhibitors) and then resuspended in 2 × SDS loading buffer. The following antibodies were used for immunoblotting: anti-GFP (1:2,500, MBL international), anti-Hec1(1:1,500, Abcam), anti-tubulin (1:4,000, Sigma), anti-CENP-T (1:5,000), anti-CENP-C (1:5,000), horseradish peroxidase-conjugated anti-rabbit IgG (1:100,000, Jackson ImmunoResearch) and horseradish peroxidase-conjugated anti-mouse IgG (1:100,000, Jackson ImmunoResearch)^13^,^35^.

### Measurement of expression level of cellular EGFP fusion protein levels

To measure total EGFP expression level in the cell, we used MetaMorph to generate a sum image where each pixel value in the image was the sum of the corresponding pixel values in each image of the *z* axis stack of images through the whole-live cell. Images were acquired with the 488-channel (∼120 images separated by 200 nm along the *z* axis) as described above. For measurements, a 330 × 330 pixel square was centred on the cell in the sum image, whereas a 400 × 400 region was used to obtain surrounding background (BG) intensity (Supplementary Fig. 1). Measured values were calculated by: (integrated fluorescence intensity−BG)=integrated intensity for 330 × 330−(integrated counts for the 330 × 330—integrated counts for 400 × 400) × pixel area of the 330 × 330 region/(pixel area of the 400 × 400 region−pixel area of a 330 × 330 region). Measurements were made with MetaMorph 7.0 software (Molecular Devices) using region measurements (Supplementary Fig. 1).

### Imaging cells for obtaining kinetochore protein copy numbers

All confocal images of live HeLa cells expressing an EGFP fusion protein were recorded at 35–37 °C as described by previously^19^,^20^. Laser (Model 35 LTL 835–220; CVI Melles Griot) illumination at 488 nm was projected through a spinning disk confocal head (Yokogawa CSU-10; PerkinElmer) at 0.5 mW into the back aperture of a × 100/1.4 numerical aperture objective lens (Nikon) mounted on a TE300 stand (Nikon). Confocal images from the Yokogawa CSU-10 were acquired using 600-ms exposures and a CCD camera (OrcaAG; Hamamatsu) typically with 1 × 1 binning for an effective pixel size of 65 nm or occasionally with 2 × 2 binning for an effective pixel size of 130 nm in specimen images. Image acquisition was controlled by MetaMorph 6.1 Software (Molecular Devices). To find the *z* axis position of best focus for analysis, which means the highest integrated intensities at kinetochore spot, a through-focus series of image exposures at 200-nm steps was acquired starting just beneath the coverslip surface.

### Image analysis for counting the copy numbers for EGFP fusion proteins

Integrated fluorescence intensity (minus BG) measurements were obtained for kinetochores in live cells as described^13^. A 10 × 10 pixel region was centred on the fluorescent spot to obtain integrated fluorescence, whereas a 14 × 14 pixel region centred on the 10 × 10 pixel region was used to obtain surrounding BG intensity. Measured values were calculated by: Fi (integrated fluorescence intensity minus BG)=integrated intensity for 10 × 10 region−(integrated counts for the 14 × 14−integrated counts for 10 × 10) × pixel area of the 10 × 10/(pixel area of the 14 × 14 region—pixel area of a 10 × 10 region). Measurements were performed with MetaMorph 7.0 software (Molecular Devices) using region measurements. Note, for the analysis in Fig. 2a CENP-T–EGFP A, we used 5 × 5 and 7 × 7 pixels regions because we took these images with 2 × 2 binning. We confirmed that the results did not change between no binning images and 2 × 2 binning images.

Fi intensity measurements were corrected for photobleaching (Fic) for EGFP measurements as well as loss of intensity as a function of depth beneath the coverslip surface (Ficc)^19^. Photobleaching of EGFP in HeLa cells was on average 0.33% for each beam exposure time interval before the in-focus image of a kinetochore was acquired (Supplementary Fig. 3a). Photobleaching was insignificant and could be neglected for immunofluorescence specimens. For each cell type, kinetochore Fic was highest near the coverslip inner surface and decreased with image plane number for the 200 nm *z* axis steps (Supplementary Fig. 4b, top plots). To correct EGFP fluorescence measurements from live cells for this depth effect, we fitted a linear regression line to the Fic data, and used the frame number at the first data measurement and the slope, to correct the Fic data at higher frame numbers (Supplementary Fig. 4b, bottom plots)^19^,^20^. This produced corrected data, Ficc, whose distribution was approximately normal (Fig. 2a; Supplementary Fig. 4a) and yielded a mean value and s.d. for the population of measured kinetochore where *n*=93–909 (Table 2). This correction method produced means close to the value measured near the coverslip while providing a measure of the s.d. for each data set.

### Fluorescence of individual EGFPs

The fluorescence standard for determining protein copy numbers was the integrated fluorescence intensity minus background of individual EGFP molecules bound to the inner surface of a coverslip in PBS at pH=7.1 measured by the above instrumentation and imaging protocol to be 13±3.7 counts at best focus^19^. To test the accuracy of this value, we measured the magnitude of fluorescence for individual EGFPs at the coverslip surface by the Graham *et al*.^36^ method. We obtained a value of 14±3 counts per EGFP using our instrumentation and our standard image acquisition protocol; a value nearly identical to the one measured by the previous method^19^.

For the Graham *et al*.^36^ method, the fluorescence minus background was measured for fluorescent beads (PS-S, P7220, Molecular Probes) bound to the inner surface of a coverslip in PBS at pH=7.1 buffer with the same instrumentation and imaging protocol as used for obtaining kinetochore protein copy numbers as described above. To obtain the fluorescence counts for individual EGFPs (F-EGFP), the mean bead fluorescence (F-beads) was multiplied by the ratio of solution fluorescence for EGFP (F-EGFP solution) and beads (F-beads solution) in PBS at pH=7.4: F-EGFP=F-bead × (F-EGFP solution/EGFP concentration)/(F-bead solution/bead concentration). For solution measurements, integrated fluorescence intensity minus BG was measured using the same instrumentation as used for determining protein copy numbers except a Nikon × 20/0.5 numerical aperture objective was used with a 1-s exposure to obtain low-magnification images of fluorescence 20 μm beneath the coverslip surface in coverslip–slide perfusion chambers about 70-μm deep. EGFP (catalog no. 4999-100; Bio Vision) protein was stored at −80° C at a concentration of ∼1 mg ml^−1^ in PBS. Before the EGFP or bead solutions were perfused into the chamber, the inner surfaces were coated with casein protein (Sigma C4765-10 ml) by incubation of a 1-mg per ml solution in PBS at pH=7.4 for 5 min to prevent surface binding of fluorescent EGFP or beads^36^. The concentration of EGFP in solution was determined using a value for the extinction coefficient at 488 nm of 55,000 (Biosciences^37^). We verified the bead concentration specified by Molecular Probes by measuring the average number of beads in focus within the full-width half-maximal focal depth of a × 100 water immersion objective at about 10-μm beneath the coverslip of a bulk bead solution used for the low-magnification measurements. Our measured bead concentration was within the 10% tolerance specified by Molecular Probes. Fluorescent counts were integrated over the central 500 × 500 pixel regions of the low-magnification images of solution fluorescence after subtraction of a camera image recorded without fluorescence illumination. More than 100 fluorescence images from different regions of the perfusion chambers were obtained and mean values and s.d. calculated for both the EGFP and bead solutions.

### Specimen preparation and image acquisition for immunofluorescence

Cells were fixed by 3% paraformaldehyde at 37 °C for 10 min as described^13^. Fixed samples were permeabilized by 0.5% NP40 (Roche) or 0.5% Triton (Sigma) in PHEM buffer (120 mM Pipes, 50 mM HEPES, 20 mM EGTA, 4 mM magnesium acetate, pH 7.0), rinsed in 0.1% Triton/PHEM and incubated in 0.5% bovine serum albumin (SIGMA) or BGS (boiled goat serum) or BDS (boiled donkey serum) for 30 min at room temperature, then samples were incubated for 1 h at 37 °C with primary antibodies. Primary antibodies to CENP-T (1:2,000), CENP-C (1:2,000), GFP (1:400, MBL international or 1:200, Abcam), mCherry (1:400, Life Technologies), tubulin (1:500, Sigma), Hec1 (1:400, Abcam), Mis12 (1:1,000), Knl1 (1:20) and Dsn1 (1:1,000) were described previously^13^,^35^. Secondary antibodies were conjugated with Alexa488, Rhodamine Red-X or Cy5 (1:400, Jackson ImmunoResearch). Chromosomes in samples were stained using 4,6-diamidino-2-phenylindole (0.1 μg ml^−1^). Samples were mounted using Prolong Antifade (Molecular Probes) or home-made mounting media (20 mM Tris pH 8.0, 0.5% *N*-propyl gallate, 90% glycerol) after post fixation was performed by 3% paraformaldehyde at room temperature for 10 min and rinsing in PBS. Images of Rhodamine Red-X, Alexa488 and Cy5 were obtained at 200 nm steps along the *z* axis through the cell to produce a *z* axis image stack using the instrumentation described above for obtaining kinetochore protein copy numbers.

### Image analysis for quantitative immunofluorescence

Kinetochore-integrated fluorescence intensity (Fi) measurements for immunofluorescent samples were obtained as described above in the EGFP fusion protein counting section. Kinetochore position for siRNA target protein was determined by transferring the kinetochore position from a fluorescent kinetochore protein of another colour, which was not the target of siRNA. For example, CENP-T kinetochore positions in CENP-T-depleted cells were determined from CENP-C kinetochore positions. We measured >35 kinetochores per cell, which were randomly selected. For immunofluorescence specimens, we also corrected for depth by same method used for EGFP in live cells (Supplementary Fig. 5a), but photobleaching was insignificant. We performed all image analysis multiple times and obtained identical results (example showed in Supplementary Fig. 5b). Note, all figures are given as mean±s.d. The use of s.e. was not necessary because of the high signal to noise of the immunofluorescent kinetochore images (Supplementary Fig. 5b)

### Cold stability assay

Before fixation, cells were treated with cold (4 °C) for 10 min, followed by fixation and staining using methods described above. For kMT intensity analysis, we used the same methods for measurement of kinetochore fluorescence described above. However, the position of the 10 × 10 pixel region for measurement of cold-stable kMT fluorescence was set proximal to and poleward from a kinetochore labelled with a different colour using anti-Ndc80/Hec1 (Supplementary Fig. 8). The 14 × 14 pixel square for background was placed like in Supplementary Fig. 8.

### Transient transfection assay

To examine how the amounts of metaphase kinetochore Ndc80/Hec1, CENP-T and CENP-C depend on protein overexpression, we transiently transfected second-colour-fused proteins into stably expressing first-colour-fused protein cell lines. For example, we transiently expressed Hec1–mCherry in cells stably expressing Hec1–EGFP after depletion of the endogenous protein by siRNAi. Transient transfection was performedusing Effectene regents (Qiagen) according to the manufacturer's instructions. Kinetochore measurements for transiently transfected cells were made 48 h after transfection. We took images with various expression levels for a transiently transfected protein using the same methods described above for EGFP fusions in the stable cell lines. Then, we measured kinetochore intensities for both green (EGFP) and red (mCherry or tdTomato) channels within the cells using methods described above. The average kinetochore intensity in each cell was obtained from >35 kinetochores within a single cell.

### Statistical analysis

Statistical significance was determined using Student's *t*-test for comparison between two independent groups. For significance, *P*<0.01 was considered statistically significant.

## Additional information

**How to cite this article:** Suzuki, A. *et al*. A quantitative description of Ndc80 complex linkage to human kinetochores. *Nat. Commun.* 6:8161 doi: 10.1038/ncomms9161 (2015).

## Supplementary Material

Supplementary Information Supplementary Figures 1-8 and Supplementary Notes 1-3

## Figures and Tables

**Table 1:** Calculation of the mean levels of each EGFP fusion protein relative to control from the ratio of western blot intensity and Commossie Brilliant blue (CBB) intensity relative to control as measured from western blots and protein staining in (Fig. 1c).

	**Ratio of CBB**	**Ratio of WB**	**Ratio of WB/CBB**
Control	1.0	1.0	1.0
Hec1–EGFP	0.7	1.1	1.5
Control	1.0	1.0	1.0
EGFP–CENP-T	0.9	1.1	1.2
Control	1.0	1.0	1.0
EGFP–CENP-C	0.6	0.6	1.0

**Table 2:** Summary of mean values of measured protein copy numbers per kinetochore and per kMT at metaphase in control cells.

	**Cell**	***N***	**Average kinetochore**	**EGFP intensities/**		**Copy # (±s.d.)**		**Normalized**
	**type**	**(kinetochores/**	**intensities**	**Hec1–EGFP**				**Hec1 intensities**
		**cells)**	**(Ficc±s.d.)**	**(±s.d.)**		**Kinetochore**	**kMT**	**(IF) (±s.d.)**
Hec1–EGFP	Stable	909/23	3,171.8±424.2	1.00±0.13		244.0±31.8	14.3±1.9	0.98±0.15
EGFP–CENP-T	Stable	263/10	955.4±112.5	0.30±0.04	Average	Average	Average	0.85±0.20
CENP-T–EGFP	Stable	302/10	905.1±99.2	0.29±0.03	0.29±0.03	71.6±8.4	4.2±0.5	1.04±0.19
EGFP–LAP–CENP-T	Stable	93/3	947.9±113.8	0.30±0.04				NA
EGFP–CENP-C	Stable	295/10	2,799.6±431.8	0.88±0.14		215.4±33.2	12.6±1.9	0.99±0.30
EGFP–Dsn1	Stable	370/7	1,934.8±281.4	0.61±0.09	Average	Average	Average	0.87±0.22
Dsn1–EGFP	Stable	309/7	1,992.0±252.3	0.63±0.08	0.62±0.08	151.1±20.6	8.8±1.2	NA
Nnf1–EGFP	Stable	416/10	1,937.5±268.3	0.61±0.08				1.13±0.14
Mis12–EGFP	Stable	218/5	2,025.3±254.8	0.64±0.08				0.89±0.24
EGFP–chimera1 (CENP-C RNAi)	Stable	197/7	1,192.4±210.1	0.4±0.1		91.7±16.1	5.4±0.9	1.10±0.23

EGFP, enhanced green fluorescent protein; IF, immunofluorescence; kMT, kinetochore microtubule; NA, not applicable; RNAi, RNA interference; WT, wild type.

Mean values for Ficc (integrated kinetochore fluorescence minus background and corrected for kinetochore depth beneath coverslip and photobleaching) for EGFP at metaphase kinetochores in each cell line stably expressing an EGFP fusion protein and depleted of endogenous protein by RNAi (see the Methods section). *N* is number of kinetochores/number of cells counted. Kinetochore mean values were normalized by dividing by Ficc obtained for Hec1–EGFP cells. Mean protein copy numbers per kinetochore were obtained by dividing kinetochore Ficc by 13, the mean Ficc value for individual EGFP molecules (see the Methods section). Mean protein copy numbers per kMT at metaphase were obtained by dividing the kinetochore protein copy number by 17.1±0.6 kMT/kinetochore for metaphase HeLa cells^21^. Mean immunofluorescence levels of kinetochore Ndc80/Hec1 at metaphase for the different cell lines expressing EGFP fusion protein exhibit values close to controls (WT) HeLa cells. Ndc80/Hec1 intensity by immunofluorescence in control (WT) equal 1.0±0.16. (Supplementary Fig. 2c). Ndc80/Hec1 intensities were normalized relative to Hec1 intensities in control.

**Table 3:** Summary of kinetochore protein copy number following the protein depletions in Figs 3b (a), 4b (b) and 6b (c) based on the control values in Table 2, and the percentage of control levels from Figs 3b, 4b and 6b.

**(a)**	**Hec1**	**CENP-C**	**CENP-T**
Control	244 (100±17%)	215 (100±29%)	72 (100±15%)
CENP-T RNAi	90 (37±7%)	213 (99± 28%)	4 (5±2%)
CENP-C RNAi	100 (41±7%)	6 (3±4%)	44 (61±13%)

**(b)**	**CENP-T**	**CENP-C**	**Dsn1**	**Knl1**
Control	72 (100±23%)	215 (100±19%)	151 (100±26%)	NA (100±16%)
CENP-T RNAi	4 (5±3%)	204 (95±23%)	79 (52±11%)	NA (50±10%)
CENP-C RNAi	42 (58±12%)	15 (7±4%)	44 (29±7%)	NA (29±6%)

**(c)**	**Hec1**	**CENP-C**	**CENP-T**	**EGFP**	**KMN network proteins**	
					**Dsn1**	**Knl1**
Control	244 (100±16%)	215 (100±29%)	72 (100±15%)	0 (0±0%)	151 (100±16%)	NA (100±14%)
CENP-C RNAi	100 (41±7%)	6 (3±4%)	38 (53±12%)	0 (0±0%)	30 (20±5%)	NA (20±5%)
Chimera1 (CENP-C RNAi)	268 (110±23%)	0 (0±10%)	38* (53±12%)	92 (100±17%)	100 (66±12%)	NA (58±10%)

NA, not applicable; RNAi, RNA interference. All values=mean±s.d.

(a) Mean values of kinetochore intensities normalized by control values for CENP-T, CENP-C and Hec1 (Fig. 3b). (b) Mean values of kinetochore intensities normalized by control values for CENP-T, CENP-C, Dsn1 and Knl1 (Fig. 4b). (c) Mean values for immunofluorescence intensities at kinetochores for CENP-C, Hec1, Dsn1 and Knl1 normalized by control values in CENP-C RNAi-treated cells and EGFP–chimera1 cells (Fig. 6b).

*This value is assumed to be equal to CENP-C RNAi value (see text).
