# Supplementary material for: A quantitative description of Ndc80 complex linkage to human kinetochores
Source: Nat Commun. 2015 Sep 8;6:8161. doi: 10.1038/ncomms9161 (PMC4569735; doi:10.1038/ncomms9161)
Supplement: Supplementary Information — Supplementary Figures 1-8 and Supplementary Notes 1-3 [file ncomms9161-s1.pdf]

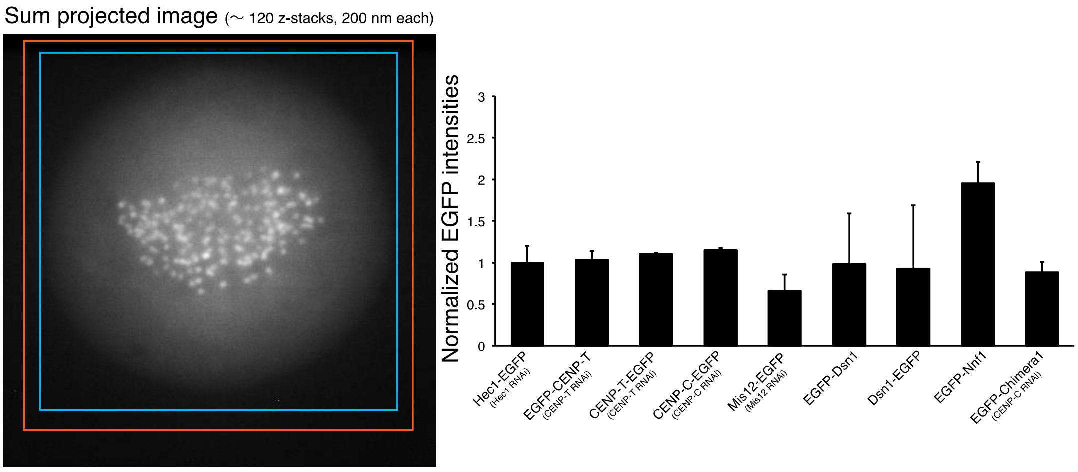

Supplementary Figure 1

Supplementary Figure 1

Properties of cell lines stably expressing kinetochore EGFP-fusion proteins after depletion of endogenous protein by RNAi. Integration of whole cell fluorescence from a z-axis image stack. The sum at each pixel position is obtained from the image stack. An example of sum-projected (488 channel) live-cell image of whole cell (approximately 120 image planes, 200 nm z-axis separation) (left). The whole cell integrated intensities of EGFP in cells stably expressing EGFP-target proteins normalized by mean value for Hec1-EGFP (right, n>20 cells in each condition). Blue box used for measurement of whole cell EGFP expression, red box used for measurement of background (see Methods). Error bars are SD from the means.

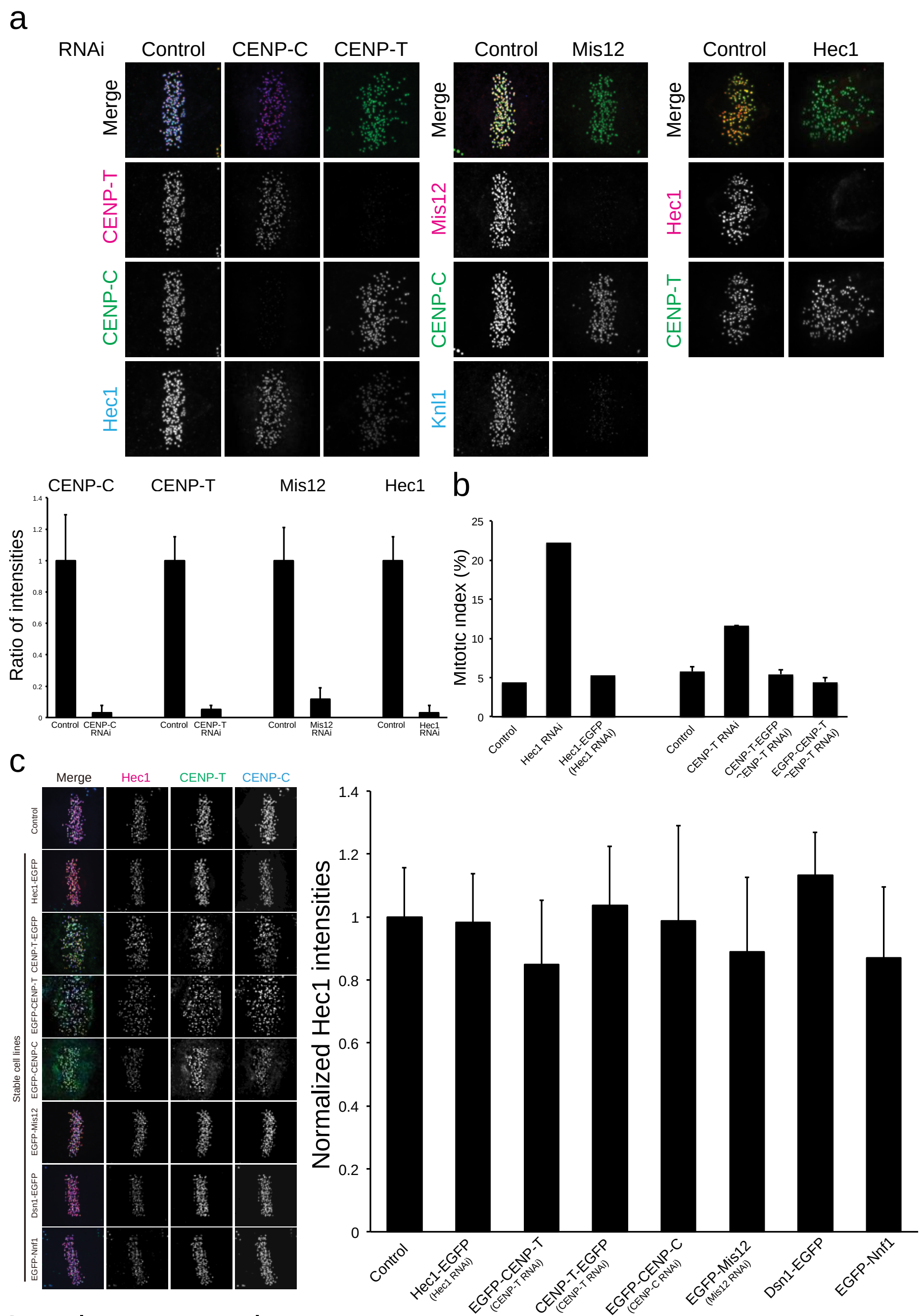

Supplementary Figure 2

Supplementary Figure 2

The level of endogenous target proteins in control, CENP-T RNAi, CENP-C RNAi, Mis12 RNAi, and Hec1 RNAi treated cells. (a) Representative images of immunofluorescence stained kinetochores with anti-Hec1, anti-CENP-T, anti-CENP-C, and anti-Mis12 in control, CENP-T RNAi, CENP-C RNAi, Mis12 RNAi, and Hec1 RNAi treated cells (Top). Mean values of kinetochore immunofluorescence normalized by control cell values for Hec1, CENP-T, CENP-C, and Mis12 for each experimental condition above (bottom). n>220 kinetochores / > 6 cells in each condition (See Methods). (b) The Mitotic index for control, Hec1 RNAi, Hec1-EGFP with Hec1 RNAi, CENP-T RNAi, EGFP-CENP-T with CENP-T RNAi, and CENP-T-EGFP with CENP-T RNAi cells. EGFP fused to target protein rescues mitotic

arrest. Error bars are SD from the means. (c) Representative three-color immunofluorescence images of kinetochores in metaphase cells stained anti-Hec1, anti-CENP-T, and anti-CENP-C (left). Mean intensities of Hec1 at kinetochores normalized by the control cell value, for cell lines expressing CENP-T-EGFP, EGFP-CENP-T, EGFP-CENP-C, and Hec1-EGFP (right). n>130 kinetochores / >3 cells in each condition. (See Methods).

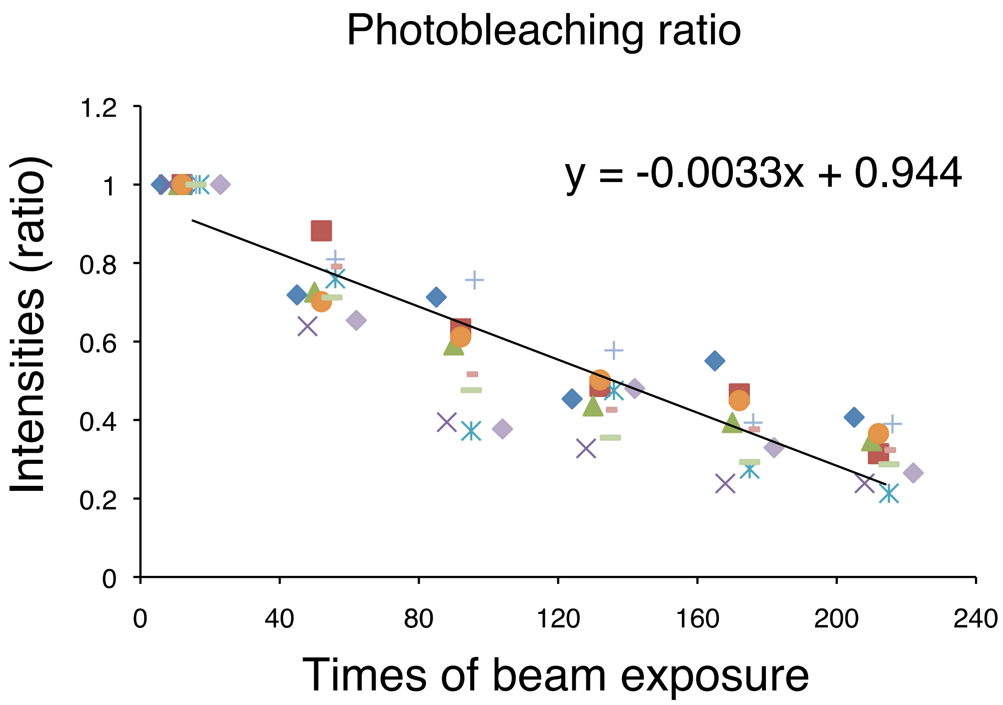

Supplementary Figure 3

Supplementary Figure 3
The photo-bleaching rate of EGFP by repeated imaging. Plot of normalized EGFP intensities (Y-axis) and times of beam exposure (X-axis). EGFP intensities in HeLa cells decreased 0.33% per laser beam exposure in our experimental condition.

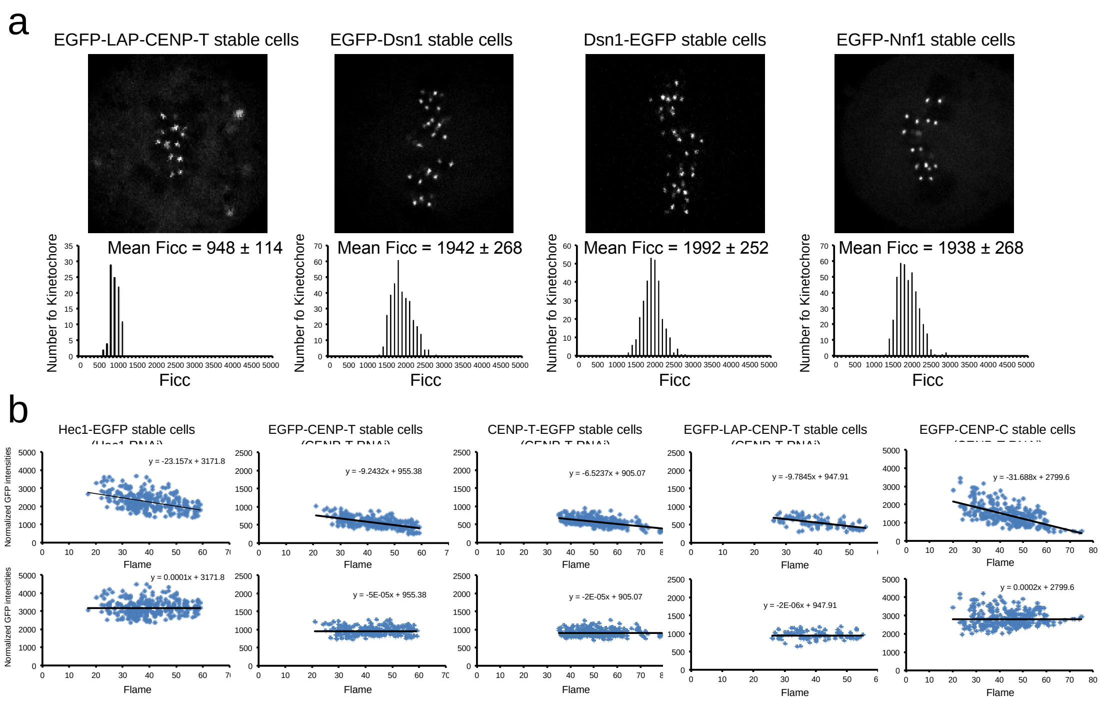

c

	Expression	Protein	Source
Hec1-EGFP	Constitutive	Human Hec1	This study
Hec1-mCherry	Constitutive	Human Hec1	This study
Hec1-tdTomato	Constitutive	Human Hec1	This study
EGFP-CENP-T	Constitutive	Human CENP-T, RNAi resistance	This study
CENP-T-EGFP	Constitutive	Human CENP-T, RNAi resistance	This study
EGFP-LAP-CENP-T	Constitutive	Human CENP-T, RNAi resistance	Gascoigne et al., 2011
EGFP-CENP-C	Constitutive	Human CENP-C, RNAi resistance	This study
EGFP-Mis12	Constitutive	Human Mis12	This study
Dsn1-EGFP	Constitutive	Human Dsn1	This study
EGFP-Dsn1	Constitutive	Human Dsn1	This study
EGFP-Nnf1	Constitutive	Human Nnf1	This study
EGFP-CT107	Constitutive	Human CENP-T, RNAi resistance	This study
EGFP-Chimera 1	Constitutive	Human CENP-C / CENP-T, RNAi resistance	This study
EGFP-Chimera 2	Constitutive	Human CENP-C / CENP-T, RNAi resistance	This study

Supplementary Figure 4

Supplementary Figure 4
The EGFP intensity measurements were corrected by depth form coverslip. (a) The representative live-cell images (upper) and the histogram plot of Ficc (lower) for EGFP-LAP-CENP-T, EGFP-Dsn1, Dsn1-EGFP, and EGFP-Nnf1 stably expressed cells. (b) The plot of EGFP intensity before depth correction (top) and after depth correction (bottom) in cells expressing Hec1-EGFP, EGFP-CENP-T, CENP-T-EGFP, EGFP-LAP-CENP-T, or EGFP-CENP-C. (c) Details for the stably expressed cell lines used in this paper (see Methods).

a

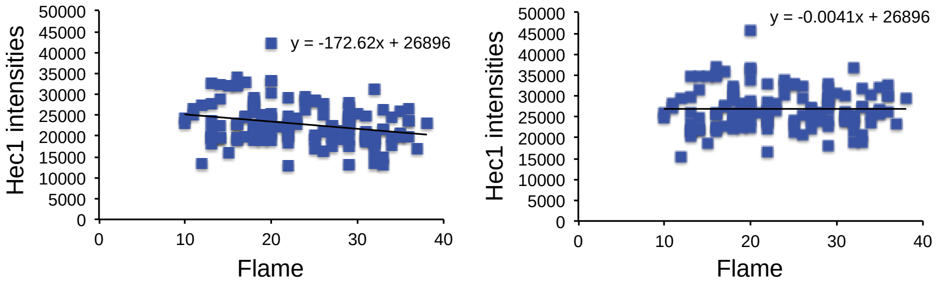

b

Experiment 1

	CENP-C		Hec1	
	Control	CENP-C RNAi	Control	CENP-C RNAi
% of intensity	100	7	100	39
S.D.	28	4	18	9
S.E.	2	0	1	1
n	224	248	187	272

Experiment 2

	CENP-C		Hec1	
	Control	CENP-C RNAi	Control	CENP-C RNAi
% of intensity	100	10	100	45
S.D.	22	4	18	11
S.E.	1	0	1	1
n	223	241	233	236

Experiment 3

	CENP-C		Hec1	
	Control	CENP-C RNAi	Control	CENP-C RNAi
% of intensity	100	5	100	42
S.D.	23	3	17	10
S.E.	1	0	1	0
n	250	217	266	278

Supplementary Figure 5

Supplementary Figure 5

Example of kinetochore immunofluorescence intensities corrected by depth beneath coverslip inner surface. (a) Plot of immunofluorescence intensity for Hec1 at kinetochores (Fi) versus frame number in a z-axis stack of images where the first image was taken at the coverslip inner surface (left). Plot after correction for depth beneath the coverslip (right). (b) An example of quantitative immunofluorescence analysis for changes in kinetochore protein concentration. Three independent experiments show identical results. The standard errors (SE) are typically around zero to 2% in our assay.

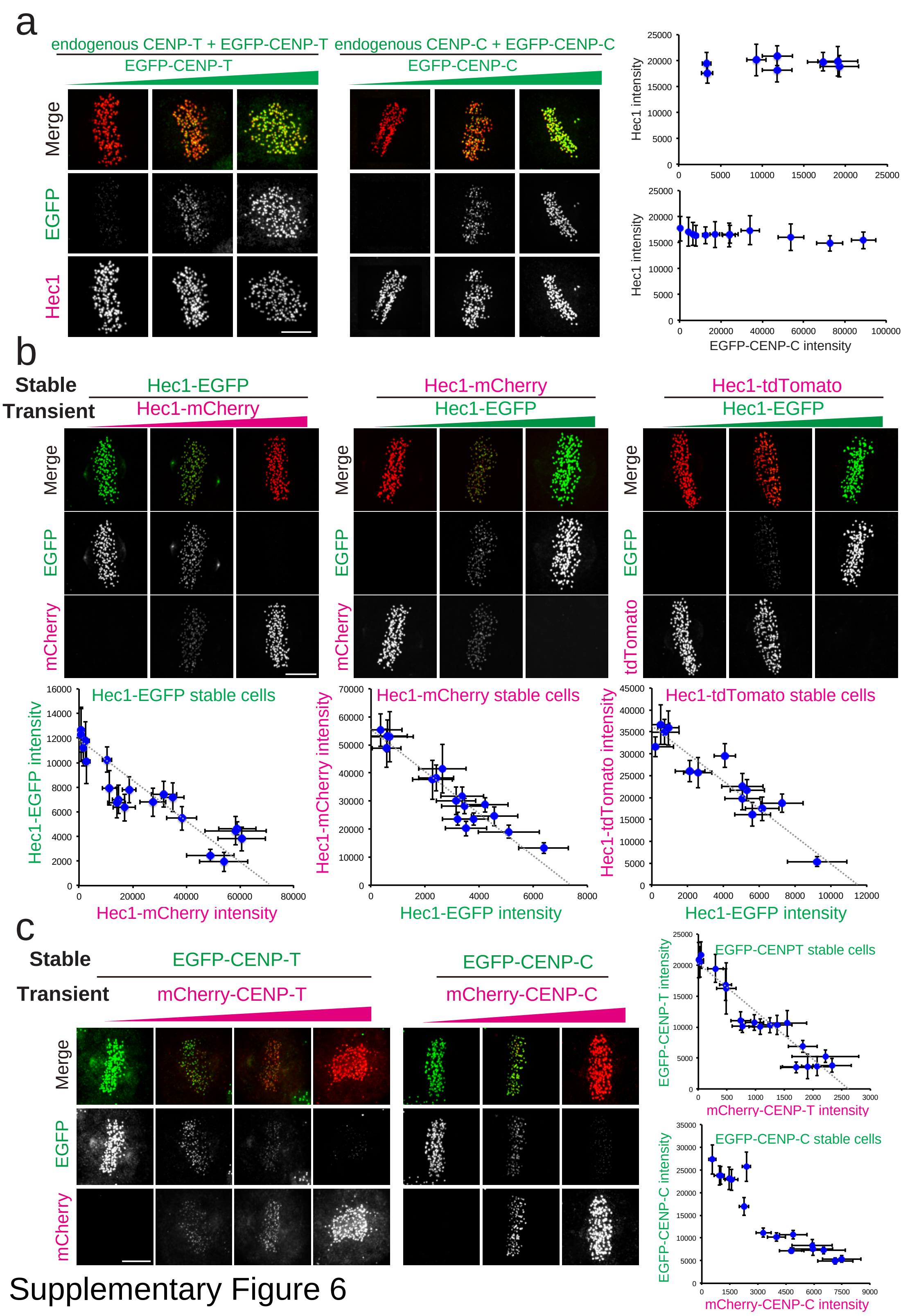

Supplementary Figure 6

Kinetochores have limited number of binding sites that are saturated at metaphase for Hec1, CENP-T, and CENP-C. (a) Mean kinetochore intensity for Hec1 remains constant (right) in HeLa cell lines with increasing overexpression of EGFP-CENP-T or EGFP-CENP-C as seen in representative two-color

immunofluorescence images of kinetochores stained with anti-GFP and anti-Hec1 in stably expressed EGFP-CENP-T cells or stably expressed EGFP-CENP-C cells (left). (b) Mean kinetochore intensity in stably expressing cells with fluorescently labeled Hec1 (-EGFP or -mCherry or -tdTomato) decreases in proportion to the level of a transiently expressed second labeled Hec1 (-mCherry or -EGFP, respectively). Representative two-color immunofluorescence images of kinetochores stained with anti-GFP or anti-mCherry or anti-tdTomato (top). Plots of the decrease in kinetochore intensity for the original labeled Hec1 with increase in the level of kinetochore Ficc of the transiently transfected second labeled Hec1 (bottom). (c) Mean kinetochore intensity in stably expressing cells with fluorescently labeled EGFP-CENP-T or EGFP-CENP-C decreases in proportion to the level of transiently transfected mCherry-CENP-T or mCherry-CENP-C respectively. Representative two-color immunofluorescence images of kinetochores stained with anti-GFP or anti-mCherry (left) and plots of mean kinetochore intensity for EGFP verses mCherry (right) (top). Plots of the decrease in mean kinetochore intensity for the original labeled CENP-T or CENP-C with increase in the level of kinetochore intensity of the transiently transfected second labeled CENP-T or CENP-C (bottom). The plotted data points in (b) and (c) represent the average kinetochore Ficc intensity measured for greater than 35 kinetochores in each cell. Error bars are SD from the means. Kinetochore measurements for transiently transfected cells were made 48 hours after transfection.

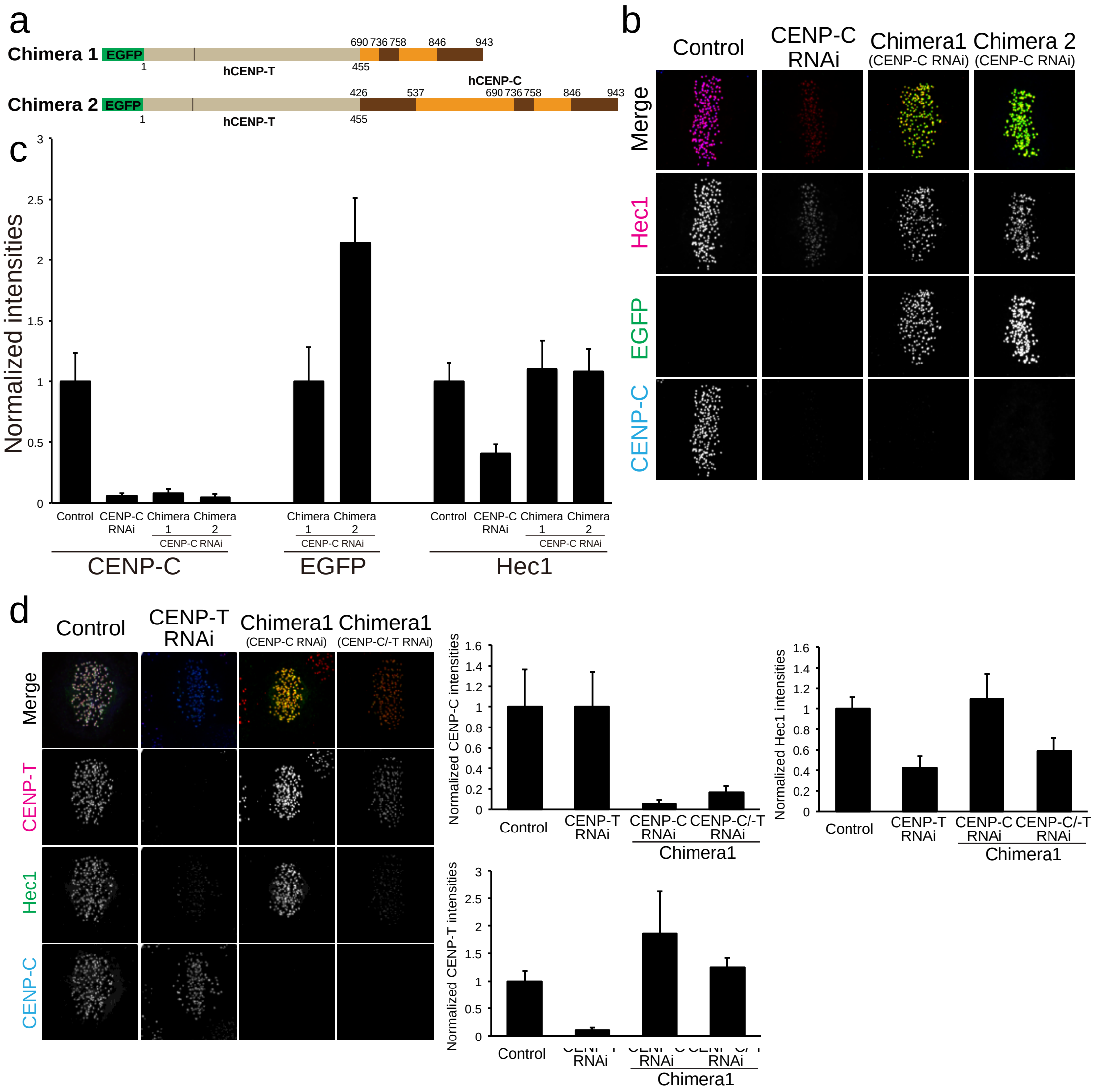

Supplementary Figure 7

Supplementary Figure 7

Chimera2 concentration at kinetochores is similar to the level of CENP-C. (a) Schematic depiction of the domain organization of Chimera1 (EGFP fused to CENP-T(1-455 aa) domain and CENP-C (690-943 aa)) and Chimera2 (EGFP fused to CENP-T(1-455 aa) domain and CENP-C (426-943 aa)). (b) Representative three-color immunofluorescence images of kinetochores stained with anti-Hec1, anti-GFP, and anti-CENP-C in control, CENP-C RNAi, Chimera1 with CENP-C RNAi, and Chimera2 with CENP-C RNAi treated cells. n>250 kinetochores / > 6 cells in each condition. (c) The mean kinetochore intensities of Hec1, EGFP, and CENP-C in each condition of (b). Hec1 and CENP-C intensities were normalized by mean control levels. EGFP intensities were normalized by mean EGFP-Chimera1 levels. (d) Representative images of three-color immunofluorescence stained kinetochores with anti-Hec1, anti-CENP-T, and anti-CENP-C in control, CENP-T RNAi, Chimera1 with CENP-C RNAi, and Chimera1 with CENP-C/-T RNAi treated cells. The mean kinetochore intensities of Hec1 (n>300 kinetochores / > 8 cells), CENP-T (n>150 kinetochores / > 4 cells), and CENP-C (n>140 kinetochores / > 3 cells) normalized by mean control levels in each condition of (d, left). Error bars are SD from the means.

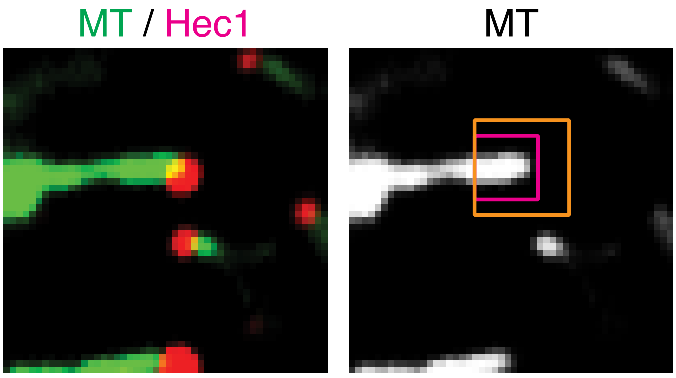

Supplementary Figure 8

Supplementary Figure 8
Measurement methods for MT intensities in cell treated with cold. Representative two-color immunofluorescence staining with anti-tubulin and anti-Hec1 in cell treated with cold (see Methods). Red-square encloses measured region for kinetochore fiber proximal to kinetochore marked by anti-Hec1 and orange-square was for background measurements.

TN: Number of Ndc80 complex links to CENP-T
CN: Number of Ndc80 complex links to CENP-C
NT: Total number of Ndc80 complex

$$NT = TN + CN = 244 \quad \text{Eqn. 1 (Ndc80 total)}$$

$$0.05\ TN + 0.99\ CN = 0.37\ NT \quad \text{Eqn. 2 (CENP-T RNAi)}$$

$$0.61\ TN + 0.03\ CN = 0.41\ NT \quad \text{Eqn. 3 (CENP-C RNAi)}$$

Recruited by	Eqn.1&2 CENP-C	Eqn.1&2 CENP-T	Eqn.1&3 CENP-C	Eqn.1&3 CENP-T
Ndc80/Hec1 molecules per KT	83	161	84	160
1 CENP-T recruits (Ndc80/Hec1 molecules)	2.2		2.2	
% of CENP-C recruiting Ndc80/Hec1	39%		39%	

Estimated number of molecules per kinetochore (Table 2)

NT: Total number of Ndc80 complex

$$NT = \text{CENP-T} + \text{Mis12 complex} = 223 \quad \text{Eqn. 4 (Ndc80 total)}$$

Recruited by	Eqn.4&2 CENP-C	Eqn.4&2 CENP-T	Eqn.4&3 CENP-C	Eqn.4&3 CENP-T
Ndc80/Hec1 molecules per KT	76	147	77	146
1 CENP-T recruits (Ndc80/Hec1 molecules)	2.0		2.0	
% of CENP-C recruiting Ndc80/Hec1	35%		36%	

Estimated number of molecules per kinetochore (Table 2)

Supplementary Note 1

Supplementary Note 1
Calculations of the stoichiometry of Hec1 recruitment to kinetochores by CENP-T and CENP-C based on protein copy numbers in Table 2 and protein depletion data Table 3(a) as described in the text.

TD: Number of Dsn1 related to CENP-T
CD: Number of Dsn1 related to CENP-C
DT: Total number of Dsn1

DT =TD + CD = 151 Eqn. 1

0.05 TD + 0.95 CD = 0.52 DT Eqn. 2 (CENP-T RNAi)

0.58 TD + 0.07 CD = 0.29 DT Eqn. 3 (CENP-C RNAi)

Eqn.1&2

TD = 0.48 DT
CD = 0.52 DT

Eqn.1&3

TD = 0.44 DT
CD = 0.56 DT

TK: Number of Knl1 related to CENP-T
CK: Number of Knl1 related to CENP-C
KT: Total number of Knl1

KT = TK + CK Eqn. 4

0.05 TK + 0.95 CK = 0.50 KT Eqn. 5 (CENP-T RNAi)

0.58 TK + 0.07 CK = 0.29 KT Eqn. 6 (CENP-C RNAi)

Eqn.4&5

TK = 0.50 KT
CK = 0.50 KT

Eqn.4&6

TK = 0.44 KT
CK = 0.56 KT

	Eqn.1&2		Eqn.1&3	
Recruited by	CENP-C	CENP-T	CENP-C	CENP-T
Dsn1 molecules per KT	79	72	86	65
1 CENP-T recruits (Dsn1 molecules)	1.0		0.96	
% of CENP-C recruiting Mis12 complex	37%		40%	

Estimated number of molecules per kinetochore (Table 2)

Supplementary Note 2

Supplementary Note 2
Calculation of the stoichiometry of Dsn1 (left) and Knl1 (right) recruitment by CENP-T and CENP-C based on protein copy numbers in Table 2 and protein depletion data in Table 3(b) as described in the text.

TN: Number of Ndc80 complex links to endogenous CENP-T
CTCN: Number of Ndc80 complex links to Chimera1
NT: Total number of Ndc80 complex

NT = TN + CTCN = 268 Eqn. 1 (Ndc80 total in Chimera cells)
TN + CTCN = 38 + 92 =130 Eqn. 2 (total N-terminus of CENP-T in Chimera1 cells with CENP-C RNAi)
From Eqn.1 & Eqn.2
Eqn.1 / Eqn.2 = 268 / 130 = 2.06
1 N-terminus of CENP-T recruits 2.06 Ndc80/Hec1 molecules

Supplementary Note 3

Supplementary Note 3
Calculation of the stoichiometry of Hec1 recruitment by CENP-T and EGFP-Chimera1 based on protein copy numbers in Table 2 and protein depletion data in Table 3(c) as described in the text.
